# Electrochemical and X-ray structural evidence of multiple molybdenum precursor candidates from a reported non-aqueous electrodeposition of molybdenum disulfide†

**DOI:** 10.1039/d3ra04605b

**Published:** 2023-11-01

**Authors:** Tanner George, Christa L. Brosseau, Jason D. Masuda

**Affiliations:** a Department of Chemistry, Saint Mary's University Halifax Nova Scotia Canada B3H 3C3 Tanner.George@smu.ca Jason.Masuda@smu.ca

## Abstract

A published report of electrodeposited molybdenum(iv) disulfide microflowers at 100 °C in the ionic liquid *N*-methyl-*N*-propylpiperidinium bis(trifluoromethane)sulfonimide (PP13-TFSI) from 1,4-butanedithiol and the concentrated filtrate from a reaction mixture of molybdenum(vi) trioxide and ethylene glycol could not be reproduced reliably, affording numerous uniquely coloured reaction mixtures that precipitated a variety of crystalline molybdenum coordination complexes. Further attempts to use the same two of these filtrates to electrodeposit molybdenum(iv) disulfide from 0.1 M PP13-TFSI in tetrahydrofuran with 1,4-butanedithiol at room temperature were unsuccessful. Various crude reaction mixtures grew crystals of different identity from eight attempts to synthesize the reported molybdenum-precursor. Single crystal X-ray diffraction (SC-XRD) offered insight into a wide range of structural features from four candidate paramagnetic precursor compounds, including a novel organomolybdenum cluster. Electrochemical studies of the various molybdenum-precursor filtrates, ethylene glycol, and 1,4-butanedithiol were conducted in 0.1 M PP13-TFSI in tetrahydrofuran, offering insight into differences between preparations of the molybdenum-precursor and interference between ethylene glycol and 1,4-butanedithiol on platinum working electrodes. Molybdenum(iv) disulfide electrodeposition attempts included cyclic voltammetry and chronoamperometry on platinum and glassy carbon working electrodes, which led to either no deposited material, or molybdenum, carbon, oxygen, and sulfur containing amorphous and non-homogenous deposits, as indicated by SEM-EDS analysis.

## Abstract 2

Various molybdenum complexes were identified reacting MoO_3_ and ethylene glycol, and both X-ray crystallography and electrochemistry provide a better understand of this complex, yet crucial reagent used for the 1st reported MoS_2_ electrodeposition.

## Graphical abstract

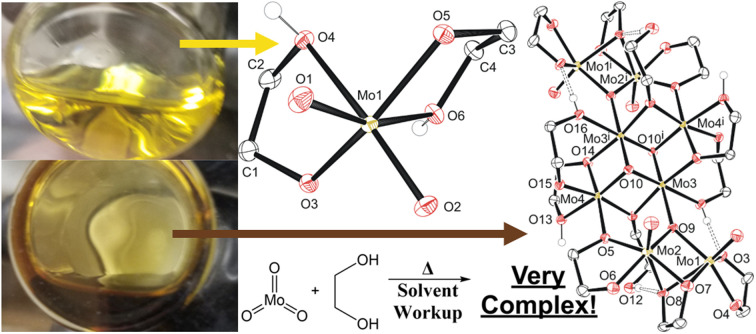

## Introduction

Crystalline MoS_2_, either pure or as a composite involving other materials such as ZnO,^2,3^ FeS_2_,^4–8^ Fe_3_O_4_,^9^ and Au,^10,11^ has shown promise for an array of diverse applications including use in supercapacitors and transistors,^4,12,13^ catalyzing the hydrogen evolution reaction (HER) and solar energy harvesting material,^1,7,8,14–20^ and environmental remediation.^3,9^ Use of 2D-MoS_2_ as a substrate for Surface Enhanced Raman Spectroscopy (SERS)^2,10,21–23^ has recently emerged, offering the potential of widespread detection of important analytes, including pesticides, biomarkers, or drugs, for agricultural, biomedical, and forensic industries.^12^

Molybdenum disulfide has multiple polymorphs (tetragonal (*D*_3d_) = 1T, hexagonal (*D*_3h_) = 2H, rhombohedral (*C*^5^_3v_) = 3R) with conducting (1T) and semiconducting (2H, 3R) properties.^14^ The semiconducting forms of MoS_2_ highlight a very important property regarding the morphology of MoS_2_; monolayers possess a direct band gap despite the bulk material possessing an indirect band gap. This different optical response results in monolayer molybdenum disulfide (2D-MoS_2_) promoting the Raman scattering of incident radiation through charge transfer to the molecule upon irradiation with an estimated Raman enhancement factor of ∼3 × 10^5^ which is comparable to values reported for silver and gold.^14,24,25^ Techniques of preparing 2D-MoS_2_ include chemical vapor deposition,^25,26^ sonication,^27^ alkali metal exfoliation,^17^ and electro-ablation of mechanically deposited bulk MoS_2_.^13^ Electrochemical methods can produce thin films of deposited amorphous MoS_*x*_ (2 < *x* < 3) which, after annealing under a sulfur or argon atmosphere at 500 or 550 °C, attain the desired crystallinity as MoS_2_.^28,29^

Electrochemical deposition of thin films of crystalline MoS_2_ with controlled thickness, without the need for annealing, is highly desired. Murugesan *et al.*^1^ reported the electrodeposition of microflowers of pure MoS_2_ by chronoamperometric deposition at −2.7 V at 100 °C for 5–10 minutes (highlighting the need for higher temperatures to achieve the reaction) within the ionic liquid *N*-methyl-*N*-propylpiperidinium bis(trifluoromethyl)sulfonamide (PP13-TFSI) using precursors, 1,4-butanedithiol for sulfur, and MoO_3_ + ethylene glycol for molybdenum.^1^ Underpotential deposition of thin films like that of copper suggest a lower voltage may be needed to produce uniform deposits compared to the 3D microflowers, however, since MoS_2_ is a metal chalcogenide rather than a pure metal, this may prevent deposition without overpotential.^28,30^ Other Mo/S sources including ammonium tetra thiomolybdate ((NH_4_)_2_MoS_4_)^28,29^ and ammonium thiodimolybdate ((NH_4_)_2_Mo_2_S_12_)^31^ have led to amorphous thin films upon electroreduction, rather than the desired thin crystalline MoS_2_. The report by Murugesan *et al.* was and remains the only reported direct electrodeposition of crystalline MoS_2_ to our knowledge, which is what led to the attempt to reproduce the methods. Our intention for replicating this work was for a reproducible, reliable morphology control *via* electrodeposition for preparing the MoS_2_ without post-treatment. This interest is shared by many fields, including materials technology,^4,12,13^ and energy generation (solar,^20^ hydrogen^1,7,8,14–19^). Our specific initial focus of trace detection of substances using MoS_2_ as a substrate for SERS led to this current study of the process reported by Murugesan *et al.*^1^

The synthesis provided by Murugesan *et al.* to prepare the MoS_2_ molybdenum precursor involves refluxing a mixture of MoO_3_ with ethylene glycol under N_2_ at 194 °C, and the workup states to simply extract the final brown viscous product. These instructions are not detailed enough to readily replicate the work. Ref. 32 and 33 provided by Murugesan *et al.* for the reaction of MoO_3_ and ethylene glycol provide no mention of a reaction between these two materials, and instead focus on the preparation of similar ammonium and alkali metal salts of a dioxo-molybdenum compound with various acids, such as glycolic acid in the case of Cuin *et al.*^32^ In the original preparation provided by Murugesan *et al.*, the name “molybdenum glycolate” is given to the molybdenum precursor, despite containing no glycolic acid-based ligands such as what is used in the work by Cuin *et al.*^32^ Work by Preiss *et al.*^33^ is slightly more related to the procedure that Murugesan *et al.* report; however, the focus of the work in this reference is preparation of molybdenum carbide (Mo_2_C). This process, used to form Mo_2_C, involves heating molybdenum(vi) oxide (or molybdic acid, the hydrated form of MoO_3_ in this case) within ethylene glycol; although, a temperature of only 60 °C is used and for an unknown duration until “…the odour of ammonia could no longer be detected…” because “(the commercial molybdic acid contains ammonium molybdate)”.^33^ A valuable point was noted in this work; concentration of samples prior to saccharose addition was possible *via* heating at ∼130 °C. Concentration with heat under vacuum, followed by filtration, was treated as the means of extracting the sample, with or without use of solvents like tetrahydrofuran or dichloromethane. Multiple reactions resulted in a viscous golden liquid and concentrating the reaction mixture caused the crystallization of MoO_2_(OC_2_H_4_OH)_2_. This gave insight into an earlier literature synthesis^2^ and crystal structure^34^ related to this preparation which heats the crude MoO_3_ + ethylene glycol mixture to 150 °C to produce MoO_2_(OC_2_H_4_OH)_2_. A golden viscous liquid or colourless crystal was not used in the report of electrodeposition of MoS_2_ by Murugesan *et al.* and attempts to replicate their electrochemical synthesis of MoS_2_ were not successful, so additional syntheses were undertaken to better understand this complex reagent.

Understanding the identity of this molybdenum-precursor is necessary for understanding the irreproducible electrodeposition of crystalline MoS_2_, and the following work sheds light on the complex identity of the molybdenum compounds that form when molybdenum(vi) trioxide is heated in ethylene glycol and explores some of the electrochemistry of these reaction mixtures within an ionic liquid electrolyte (0.1 M PP13-TFSI ionic liquid in tetrahydrofuran (THF)). The three primary reasons for abandoning the pure ionic liquid solvent were that (i) the ethylene glycol solutions of the molybdenum precursor did not readily dissolve into the ionic liquid on the scales used without significant manual mixing (ii) the pure ionic liquid is relatively expensive, and to conserve the material, use as an electrolyte would be ideal over use as a solvent (iii) at higher temperatures, precipitation occurred when mixing the molybdenum precursor, the ionic liquid, and the dithiol.

The purpose of these initial studies can be broken down into three aspects highlighting the need for optimization and further understanding of this specific electrochemical deposition of MoS_2_; (i) to explore the identity of the poorly characterized yet necessary molybdenum-precursor *via* single crystal X-ray diffraction (SC-XRD), (ii) to characterize some electrochemical properties of the molybdenum-based precursors, 1,4-butanedithiol, ethylene glycol, and mixtures of these reagents on platinum or glassy carbon working electrodes in 0.1 M PP13-TFSI in THF along with the electrochemical window of this supporting electrolyte, and to (iii) attempt to electrochemically produce MoS_2_ from either pure PP13-TFSI following the work by Murugesan *et al.*, or in a solution containing 0.1 M PP13-TFSI in THF using 1,4-butanedithiol as the sulfur source reaction mixtures of MoO_3_ and ethylene glycol as the source of molybdenum.

## Experimental

### Solvents, reagents, and materials

Nitrogen (gas (99.999%) and liquid) was supplied by Air Liquide Canada Inc. (Montreal, QC, Canada). Ethanol (95% and absolute) was purchased from Commercial Alcohols (Boucherville, QC, Canada). Toluene (99.9%), acetone (99.7%), THF (HPLC grade, anhydrous), hexanes (98.5%), dichloromethane (99.9%), and chloroform (99.8%) were purchased from Fisher Chemical or Fisher Scientific (Pittsburgh, PA, USA). Anhydrous magnesium sulfate and acetonitrile were purchased from Caledon Laboratories Ltd (Armstrong Ave, Georgetown, ON, Canada). Nitric acid (69.0–70.0%) was purchased from J.T. Baker (Phillipsburg, NJ, USA) and sulfuric acid (95.0–98.0%) was from BDH. CDCl_3_ (99.8%, 0.05% v/v tetramethyl silane) was purchased from Cambridge Isotope Laboratories Inc. (Tewksbury, MA, USA). Ethylene glycol (≥99%), molybdenum trioxide (99.99%), 1,4-butanedithiol (97%), *N*-methylpiperidine (99%), 1-bromopropane (99%), 4 Å molecular sieves, pentane (99.9%), and calcined diatomaceous earth were all purchased from MilliporeSigma (Burlington, MA, USA). Activated alumina (80–200 mesh) used for drying and filtering solvents was purchased from Thomas Scientific (Swedesboro, NJ, USA). Heating was performed using a DrySyn aluminum heating block with 50–1000 mL flasks including PTFE-sealed reaction vessels, round bottom flasks, and Schlenk flasks. Potassium hydride was purchased as a 30% mineral oil dispersion from MilliporeSigma and, within a glovebox (due to pyrophoric hazards), was washed four times with pentane then dried *in vacuo* to give a free-flowing powder. Alumina, calcined diatomaceous earth, and molecular sieves were pre-dried in a 140 °C oven for a minimum of one week before being dried at 300 °C *in vacuo* in a half-filled round-bottom flask covered in aluminum foil within an aluminum block. Solvents (toluene, pentane, THF) were purified using an Innovative Technology solvent purification system. Solvents (pentane, toluene, THF, hexanes, benzene (99.8%)) were then dried using KH for 24–78 hours and subsequently filtered through dry alumina and stored over 4 Å molecular sieves (∼1/10th the volume of solvents). The KH contaminated alumina was mixed thoroughly, allowed to dry in the glovebox until free flowing, and was then removed from the glovebox and allowed to slowly decompose in a large stainless-steel bowl over several days with water being very gradually added after a day and mixed to allow for all KH to decompose gradually and safely before disposal in solid waste.

### Equipment, instruments and analytical methods

#### Electrochemical experiments

Electrochemical measurements were performed using a Wavenow USB potentiostat with Aftermath software (Version 1.5.9888, Pine Research Instrumentation, Durham, NC, USA) to control the electrochemical parameters. Origin 2018 software (OriginLab Corporation, Northampton, MA, USA) was used for all electrochemical data analysis. All electrodes and electrochemical cells were purchased from Pine Research Instrumentation (Durham, NC, USA). Electrochemical cleaning of platinum screen-printed electrodes (Pt SPE) with a built in Pt counter electrode (CE) and Ag/AgCl reference electrode (RE) was accomplished *via* potential cycling at 300 mV s^−1^ from −0.2 V to +1.0 V in an electrolyte solution containing N_2_ purged 0.5 M H_2_SO_4_ in Milli-Q water in a 20 mL scintillation vial. Dry N_2_ gas was used to purge the supporting electrolyte while all Pt SPE's were cleaned, and prior to non-aqueous experiments, they were dried under a stream of dry N_2_ gas. All aqueous electrochemistry employed an external Ag/AgCl RE, and all non-aqueous electrochemistry utilized a silver wire inserted in a sealed glass tube with a ceramic frit within either pure PP13-TFSI or 0.1 M PP13-TFSI in THF solution as the electrolyte. Electrochemical experiments were performed in dry, low-O_2_ (<100 ppm) conditions under an atmosphere of N_2_ within an mBraun Labmaster SP inert atmosphere glovebox in a low volume three electrode cell or a compact voltammetry low volume cell kit (Pine Research Instrumentation). All non-aqueous electrochemical equipment and the laptop were used within the glovebox. Two distinct three-electrode set-ups were used for non-aqueous electrochemical experiments for either glassy carbon or platinum working electrodes. The Pt SPE WEs were countered by an internal platinum CE and referenced to Ag/Ag^+^ within a compact low volume voltammetry cell. When using a 3.0 mm diameter glassy carbon WE, a 0.5 mm diameter, 65 mm length platinum wire CE shrouded in PTFE within an epoxy tube was used with the same Ag/Ag^+^ RE within a low volume three electrode electrochemical cell. Glassware was dried at 140 °C overnight prior to synthetic experimentation. Synthetic reactions were conducted in common glassware or Schlenk flasks if outside the glovebox. Non-air sensitive chemistry was performed in typical glassware such as round bottom flasks or 20 mL scintillation vials open to air. Milli-Q water (“ultrapure water”, ≥18.2 MΩ cm) was used to prepare all aqueous solutions and for rinsing electrochemical glassware and electrodes.

The glassy carbon working electrode was cleaned by polishing with 5 μm alumina on a wet polishing pad, rinsing with water followed by acetone, and finally drying in a vacuum desiccator overnight. Ceramic screen-printed electrodes (SPE, Pt WE) were cleaned by immersion in concentrated sulfuric acid for ∼20 minutes, rinsing with ultrapure water, immersion in 10–20% HNO_3_ for ∼20 min, and rinsing again with ultrapure water. This was followed by immersion in a 20 mL scintillation vial in 0.5 M H_2_SO_4_ made up with ultrapure water. In all investigations the counter electrode was Pt (either wire or as part of the SPE) and the reference was an Ag/AgCl electrode for aqueous studies, while for non-aqueous studies an Ag/Ag^+^ RE was used. For aqueous and non-aqueous electrochemical studies, reported potentials are *versus* Ag/AgCl or Ag/Ag^+^, respectively, unless otherwise noted. The working electrode was cycled in 0.5 M H_2_SO_4_ from −0.2 V to +1.0 V at a rate of 250–300 mV s^−1^ until a cyclic voltammogram consistent with clean platinum was obtained (Fig. S12†). In this work, five different Pt SPEs were used for efficient experimentation. As presented in Fig. S12† each of the five Pt SPE's used have slightly varying electrochemically active surface areas but are consistent with literature.^63^ Pt SPE#5 has the lowest area, followed by Pt SPE #1, and Pt SPE #2–4 have relatively similar electrochemically active surface areas.

#### NMR (nuclear magnetic resonance) spectroscopy

Analysis was conducted using a Bruker Avance 300 MHz NMR spectrometer and analyzed with TopSpin 4.1.3. Trace amounts of non- or partially deuterated solvent, or tetramethyl silane (when present) were used as internal references for ^1^H and ^13^C NMR spectra.^35^ For ^19^F NMR spectra no external calibration was performed since analysis of the purity of the ionic liquid was the primary focus (single ^19^F resonance).

#### Elemental analysis

Elemental analysis was performed using a PerkinElmer 2400 II series Elemental Analyzer in the Centre for Environmental Analysis and Remediation (CEAR) facility at Saint Mary's University. Air sensitive samples were prepared within a glovebox, sealed in 20 mL scintillation vials, and rapidly weighed/inserted into the analyzer offering at most ∼2 minutes of possible air exposure after opening the vial. The only non-air sensitive sample was compound 1 and it was analyzed open to air regularly.

#### Scanning electron microscopy with energy dispersive spectroscopy

Analyses were performed using a TESCAN MIRA 3 LMU Schottky Field Emission Scanning Electron Microscope, equipped with an Oxford Instrument X-max 80 mm^2^ EDS system, using the latest Silicon Drift Detector (SDD) technology to identify chemical phases. The very bright area in the images is associated with charging caused by the poor conductivity of the surface, since the glassy carbon working electrode is too long to fit into the sputter coater, and carbon tape alone was not sufficient to dissipate the charge.

#### Single crystal X-ray diffraction

Measurements involved selection of a suitable single crystal which was mounted to the tip of a MicroLoop with Paratone-N oil. Measurements were made on a Bruker D8 VENTURE diffractometer equipped with a PHOTON III CMOS detector using monochromated Mo Kα radiation (*λ* = 0.71073 Å) from an Incoatec micro-focus sealed tube at 100–150 K with APEX4 user interface.^36^ The initial orientation and unit cell were indexed using a least-squares analysis of the reflections collected from a complete 180° phi-scan with 1° per frame. For data collection, a calculation strategy was developed to maximize data completeness and multiplicity in a reasonable amount of time using the Bruker APEX4 software suite.^36^ The crystal to detector distance was set to 4 cm. Data collection, unit cell refinement, data processing, and multi-scan absorption correction were applied using the APEX4 software package.^36–38^ The structures were solved using SHELXT^39^ and all non-hydrogen atoms were refined anisotropically with SHELXL.^40^ PLATON^41^ graphical menu was used *via* OLEX2 (ref. 42) to refine and determine the validity of crystal structure determinations. Crystal structure diagrams were prepared using Ortep3.^43^ To aide in solving the structure of compound 4 initially, the solvent molecules were removed temporarily within OLEX2 (ref. 42) using the solvent mask based on work by Spek and Van Der Sluise.^44^ Hydrogen atom positions were idealized and ride on the atom to which they were attached except for hydrogen atoms bound to heteroatoms or if participating in hydrogen bonding. The final refinement included anisotropic displacement factors on all non-hydrogen atoms.

### Synthesis of molybdenum-based precursors

**Fig. 1:**
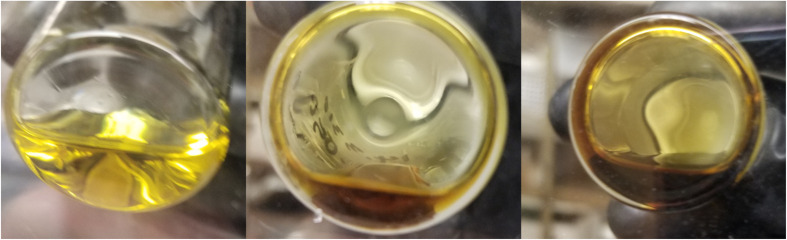
Images of B3a (gold, left), B5a (golden-brown, middle), and B6a (brown, right) crude reaction mixtures of MoO_3_ and ethylene glycol after heating, concentration *in vacuo*, and filtration through diatomaceous earth.

While many reproducibility issues were encountered preparing this precursor, the three most relevant solutions which were used as the molybdenum source are shown in Fig. 1, and details of syntheses for these three precursor solutions follow, with additional preparations relevant to this work included in the ESI.† Based on SC-XRD analysis, compounds 1 (MoO_2_(OC_2_H_4_OH)_2_), 3 (Mo_4_O_20_C_16_H_36_), and 4 (Mo_8_O_32_C_24_H_54_·2(C_2_H_4_(OH)_2_)) crystallized from the gold coloured (B3a), golden-brown coloured (B5a), and brown coloured (B6a) reaction mixtures, respectively. Details relating to reaction mixture numbering/naming are included as shown below in Tables 1, S1 and S2.† Details of the three precursor syntheses relevant to later electrochemical experiments are as follows:

**Table 1:** Final products derived from 8 reactions of MoO_3_ and ethylene glycol with varying chemical workup. Liquids indicated with (*) used within electrochemical experiments as a molybdenum-precursor

Rxn.	Liquid (B#x)	Crystal (C#x)	Solid (D#x)
A1	B1a gold viscous liquid	C1a – compound 1	N/A
A2	B2a green viscous liquid (THF)	C2a – compound 2, C2b – compound 1	D2a – dried compound 2
A3	B3a gold viscous liquid*	C3a – compound 1, C3b – compound 5	N/A
A4	B4a blue liquid	C4a – compound 6	D4a black solid, D4b blue solid (B4a dried)
A5	B5a golden-brown viscous liquid*	C5a – compound 3	D5a, D5b
	B5b golden-brown viscous liquid		
A6	B6a brown viscous liquid*	C6a – compound 4	D6a
A7	B7a gold viscous liquid	N/A	N/A
A8	B8a gold viscous liquid	N/A	D8a blue solid (B8b dried)
	B8b blue liquid		

#### Third synthesis of the molybdenum-precursor – A3

In a 100 mL round bottom flask 0.345 g MoO_3_ and 40 mL ethylene glycol were heated under vacuum to 185 °C with the distillation apparatus attached. ∼5 mL of colourless liquid was removed. Vacuum ceased and the reaction continued to warm to 194 °C under N_2_. After 10 minutes at 194 °C, a clear green solution was present which persisted for the first 35 minutes of the reaction, and after 1 hour a clear golden solution was present with a small amount of black solid. The heat was reduced to 110 °C and the subsequent vacuum distillation afforded little ethylene glycol after 2 hours, so the temperature was raised to 120 °C for 30 minutes and then 130 °C. An hour later, ∼5 mL of a golden viscous liquid with a fine black solid remaining in the flask. The whole distillation apparatus was cooled and sealed under vacuum and transferred to the glovebox. The golden viscous liquid with black solid was filtered through a diatomaceous earth pipette filter to remove the black solid, and the total mass of the golden viscous liquid (B3a, Fig. 1) was 3.1782 g and storage at room temperature in a glovebox produced crystals (C3a) over 10 months. These were identified by SC-XRD to be MoO_2_(OC_2_H_4_OH)_2_. Isolation afforded ∼0.1 g white crystals. An additional crystalline material (compound 5, [PP13]_2_[Mo_6_O_19_]) from use of B3a for attempting electrodeposition was isolated (C3b) that is shown in Fig. S11.†

#### Fifth synthesis of the molybdenum-precursor – A5

To a 250 mL Schlenk flask 0.544 g MoO_3_ and 70 mL ethylene glycol were heated to 191–193 °C for 1 hour under nitrogen. As the mixture began to heat to 180 °C, a green solution was present turning golden greenish black by 190 °C. The mixture reached 194 °C in 20 minutes and the heating continued for 1 hour. The reaction was cooled to 100 °C and a 0.5–2 mL sample was taken and placed in a vial with heavy nitrogen flow, then sealed in a 100 ppm O_2_ glovebox at room temperature. A vacuum distillation was started following this and was vigorous until ∼5–10 mL was removed. The solution was dark green at this point, and the temperature was increased to 115 °C for 2 hours until about 5 mL dark greenish brown liquid with black solids was present. This solution was filtered through a diatomaceous earth pipette filter affording 4.347 g of a thick golden-brown viscous liquid (B5a, Fig. 1). The residue in the flask and on the filter were rinsed with 3 × 1.5 mL THF, then concentrated *in vacuo* to a thick golden-brown viscous liquid weighing 0.728 g (B5b). The pipette filter was broken so the Kim wipe, diatomaceous earth, and black solid within the base of the pipette could be stored in a vial and allowed to dry slowly of THF (D5a). Over time, solutions B5a and B5b both crystallized visually similar golden crystals. A single crystal sample from B5b was analyzed by SC-XRD and shown to be compound Mo_4_O_20_C_16_H_36_ (C5a).

#### Sixth synthesis of the molybdenum-precursor – A6

This was an attempt to repeat the largest scale golden viscous liquid synthesis (#3). In a 250 mL Schlenk flask, 0.286 g MoO_3_ and 35 mL ethylene glycol and heat to 130 °C under vacuum, removing ∼10 mL liquid. Heat was raised to 194 °C for one hour, leading to a brown solution with black solids. The temperature was then reduced to 130 °C to begin vacuum distilling the mixture. Once roughly ∼8 mL thick brown viscous liquid with black solids remained, cooling, and transferring into a glovebox (with an internal vacuum in the flask to avoid opening during transfer) followed by filtration of the solution through a diatomaceous earth pipette filter afforded 6.0383 g of a brown viscous liquid (B6a, Fig. 1) with black solid (D6a) remaining on the filter. The reaction flask and the black solid were rinsed with 4 × 2 mL THF and the black solid was analyzed by SEM-EDS (Scanning Electron Microscopy – Electro Dispersive Spectroscopy), yielding a Mo : O ratio of 1 : 2.8. The combined THF washings were discarded. In 3.5 months at room temperature in a sealed 20 mL scintillation vial in the glovebox, red crystals (C6a) formed from B6a. The bulk solution was decanted, and the crystals were left to sit in ∼1 mL of the crystallization solution since rinsing with ethereal solvents was thought to potentially dissolve or alter the small amount of material. Analysis of a single red crystal by SC-XRD revealed the structure Mo_8_O_32_C_24_H_54_·2(C_2_H_4_(OH)_2_).

## Results and discussion

### Synthesis and characterization – ionic liquid preparation

To prepare the ionic liquid solvent PP13-TFSI, Li-TFSI was used as the anion [TFSI^−^] precursor while the cation [PP13^+^] precursor PP13-Br was prepared synthetically from *N*-methyl piperidine and 1-bromopropane *via* a modified literature procedure detailed in the ESI (Scheme S1†).^45^

The cation precursors were heated to 70 °C in acetonitrile for 24 hours with PP13-Br precipitating out as a white solid in the golden liquid upon cooling. Combining PP13-Br and Li-TFSI rapidly as aqueous solutions causes precipitation of a dense (>1 g mL^−1^), slightly viscous colourless liquid within an hour of mixing. Extraction with dichloromethane, washing with ultrapure water a minimum of 3 times, and drying *in vacuo* with heat resulted in the PP13-TFSI ionic liquid used for electrochemical experiments.^1^ Nuclear magnetic resonance spectra (^1^H, ^13^C, ^19^F) of the PP13-Br, PP13-TFSI, and the 1,4-butanedithiol can be found in the ESI (Fig. S1–S8).†

### Synthesis, characterization, and discussion – molybdenum-based precursors

Multiple molybdenum-based precursors were made from MoO_3_ and ethylene glycol following a modified literature preparation^1^ that involved heating the mixture to 194 °C for 1 hour under N_2_ gas flow. Potential deviation from this literature report was in the process of extraction of the final product, as little information was provided to do so: “brown colour viscous final product was extracted after the reaction.”^1^ Generally when preparing the molybdenum-precursor, concentration *via* vacuum distillation between 110 and 130 °C to remove excess ethylene glycol and subsequent filtration through diatomaceous earth to remove insoluble material, with or without added solvents, constitute the primary means of extraction within this work. No attempt was made to utilize the un-altered solution post-heating without modification due to the use of the term “extracted”, which may be what is required for successful electrodeposition under conditions described later in this report. Detailed reaction conditions are outlined in Tables S1 and S2† and in the experimental section. Eight different syntheses of this molybdenum-precursor were attempted (A(1–8), A = molybdenum-precursor reaction, #1–8 = chronological order of reaction), with varied reaction conditions and work up affording three types of products (liquid, crystalline, and amorphous solid) which are listed in Table 1. For discussion, B = liquid samples of crude reaction mixtures that have been concentrated and filtered through diatomaceous earth, with or without alternate solvents for extraction. C = Crystalline samples which have grown from liquid samples. D = fine amorphous solids. These products gave insight into the complex nature of this reaction and the sensitivity of the coordination complexes from the reaction between MoO_3_ and ethylene glycol at high temperatures.

The visual progression during these eight syntheses evolves from mint-green powder (MoO_3_) in colourless liquid (ethylene glycol), and upon heating, dissolution begins through a brief vivid turquoise colour change into a golden green hued solution, maintaining a completely transparent, solid-free lighter golden colour if kept below 150 °C (A8) and darkening if heat is increased to 194 °C. The darker golden-green solution occurs with trace black precipitate for A (1–3, 7), and significant amounts for A (4–6) and heating the mixture to 215 °C formed primarily black precipitate for A4. Gold (B1a, 3a, 7a, 8a), green (B2a), golden-brown (B5a, B5b) and brown viscous liquids (B6a) upon *in vacuo* concentration and filtration through diatomaceous earth either neat or dissolved in dichloromethane or THF. The fourth reaction was heated beyond 194 °C, affording a hard black solid rather than a viscous liquid upon concentrating *in vacuo*.

Naming of samples follows the form: sample type-reaction number-product, so the sample B-3-a refers to the viscous liquid sample (B) from reaction A3 and it is the major liquid product (a), while C2a and C2b refer to the major and minor crystalline products of A2 isolated from crystallization of the bulk solution (C2a), or from evaporating the water washings from the glassware after reaction (C2b), respectively.

**Fig. 2:**
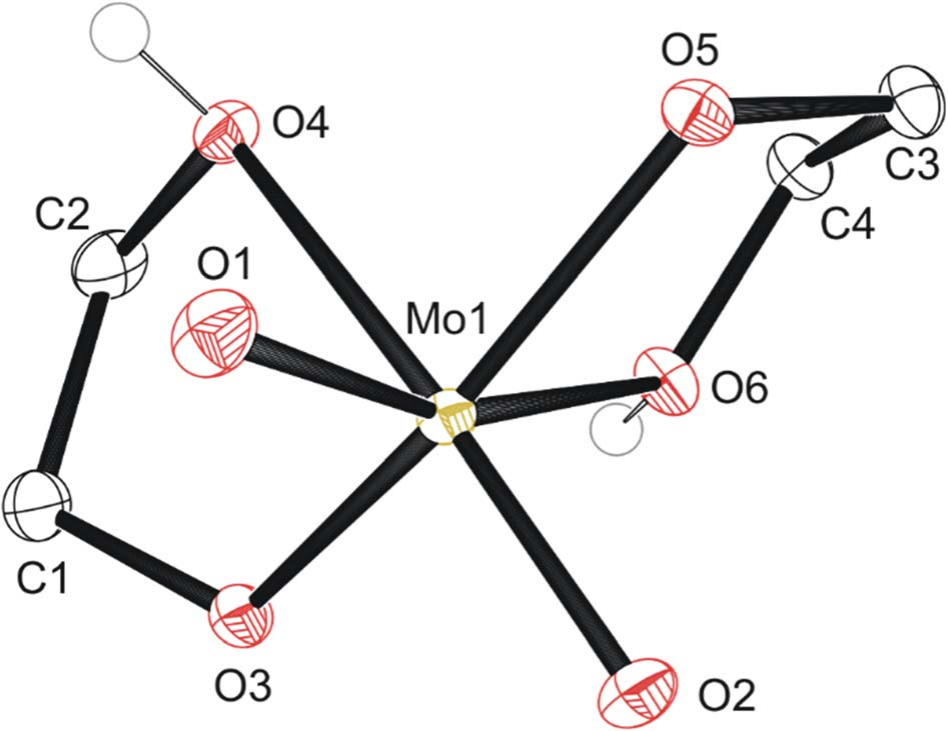
Molecular structure of compound 1 with anisotropic displacement ellipsoids projected at the 50% probability level. Hydrogen atoms have been omitted for clarity except for OH groups.

**Scheme 1:**
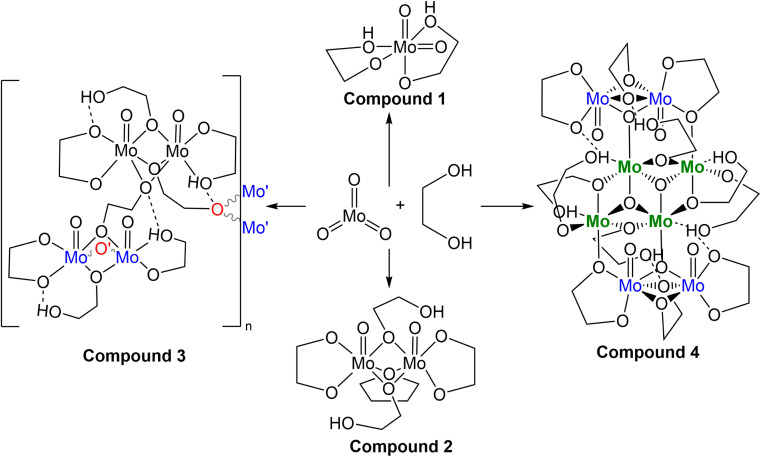
Various crystalline products isolated from the reaction between MoO_3_ and ethylene glycol.

The first, second, and third reaction mixtures A1, A2, and A3 crystallized a known molecular species, compound 1 MoO_2_(OC_2_H_4_OH)_2_ (Fig. 2), from an ethylene glycol and dichloromethane solution (C1a), ethylene glycol and water (C2b), ethylene glycol and PP13-TFSI (C1c), pure ethylene glycol (C3a), and a mix of acetonitrile and ethylene glycol (C3b). Isolating compound 1 from various mixtures and solvent systems offered insight into the original 1973 preparation^46^ and the 1975 X-ray crystallography study^34^ of this monometallic structure and the relevance of this chemical species within the MoO_3_ + ethylene glycol reaction. A diagram illustrating the hydrogen bonding in the asymmetric unit is shown in Fig. S9.† All crystalline products isolated *via* attempting this precursor synthesis are detailed in Scheme 1.

Isolation of compound 1 provided insight into workup of the MoO_3_ + ethylene glycol reaction as work in the 1970's focused on the preparation *via* similar means.^34,46^ The seventh reaction which replicated this alternate literature preparation by heating to 150 °C open to air followed by concentration *in vacuo* and addition of ethanol produced a dark blue solution rather than the expected colourless compound 1. This blue colour is thought to be due to residual water in the absolute ethanol used since hydrolysis of crude reaction mixtures, the pure MoO_2_(OC_2_H_4_OH) crystals, and the black solid D4a all afforded equally rich blue solutions in water. A series of freezing (−18 °C) and thawing (20–25 °C) cycles every few days over the course of a few weeks enabled isolation of a single tiny blue crystal (C4a – compound 6) that indicate [M]_*n*_[Mo_12_O_40_X]^*n*−^ structure by SC-XRD (X = heteroatom such as Cl, S, P, Si based on electron density where *n* = 1, 2, 3, 4 for each respectively). The cations and water positions cannot confidently be determined but are suspected to be common group 1–2 ions in tap water such as [Na]^+^ based on abundance and matching electron density at some sites. Poor data gave uncertainty regarding co-crystallized species such as the central heteroatom, cations, or water molecules; however, the general Mo_12_O_40_ structure is apparent by SC-XRD (Fig. S10†). Any reaction mixture or sample which encountered water and air for prolonged periods of time turned blue upon hydrolysis. This decomposition *via* exposure to air and moisture highlights the instability of the proposed molybdenum-precursor that was noted by Murugesan *et al.*^1^ An additional unexpected crystalline material was obtained (compound 5) through quenching an electrodeposition solution containing 1.5 mL 0.1 M PP13-TFSI in THF with 0.05 mL 1,4-butanedithiol and 0.05 mL B3a (details below). Addition of 0.5 mL 5% bleach afforded a biphasic solution with a blue bottom (thought to be aqueous) and colourless top layer (thought to be organic), along with the formation of a single ∼10–30 mg golden yellow prisms at the interface. Analysis by SC-XRD indicated the decomposition of the ionic liquid *via* anion exchange, forming [PP13]_2_[Mo_6_O_19_], compound 5 (Fig. S11†). Following the identification of compound 1 from this precursor reaction, work up of concentrated reaction mixtures with THF (A2, A5) revealed unexpected insights into the complexity regarding the reaction of MoO_3_ with ethylene glycol *via* SC-XRD analysis.

**Fig. 3:**
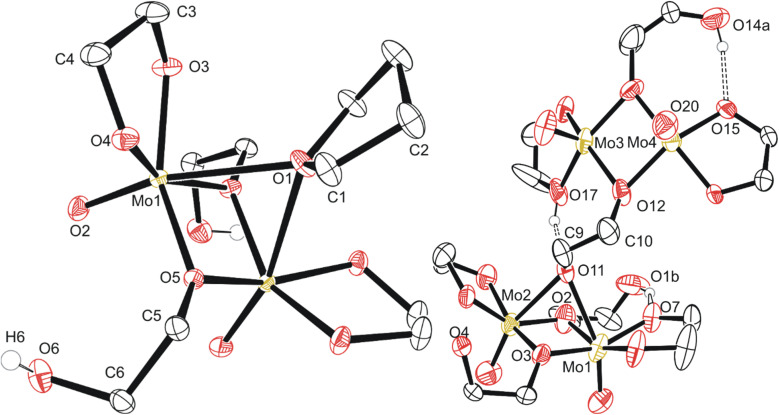
Molecular structure of compounds 2 (left, grown across central symmetry element) and 3 (right, asymmetric unit) with anisotropic displacement ellipsoids projected at the 50% probability level. Methylene CH_2_ hydrogen atoms have been omitted for clarity.

The second reaction again resulted in a golden viscous liquid upon concentration *in vacuo* of the crude reaction mixture, though extraction with dichloromethane, drying *in vacuo* again and extracting once more with THF afforded a viscous green solution upon concentration. Yellow crystals of compound 2 ((MoO(OC_2_H_4_O)(μ-OC_2_H_4_OH))_2_(μ-OC_2_H_4_)), identified by SC-XRD (Fig. 3), formed in the green viscous liquid, and, upon isolation and drying, the crystals became opaque. Elemental analysis of this material was inaccurate regarding the expected C/H% (∼1% above C, ∼2% below H) for the formula derived from SC-XRD. This synthesis was the first insight into dimerization, a process crucial for further clustering observed in reaction mixtures closer in colour to brown (A5 and A6).

Based on a Cambridge Structural Database (CSD, version 5.42 (September 2021)) search, compound 2 is the first report of crystalline molybdenum dimers with each molybdenum bound to a single oxo group (2−), an ethylene glycoxide ligand (2−), and a bridging ethylene hydroxy glycoxide (1−), although similar dimers with different ligands exist with similar bridging of the two Mo atoms by oxygen-based ligands.^47–60^ Related molybdenum dimers have had their structure reported in the CSD, specifically with each Mo atom in the dimer containing a non-bridging single oxo ligand and a bidentate O–C–C–O structured ligand. One similar non-anionic dimer features a structurally similar bidentate mono-deprotonated tetramethyl ethylene glycol and two bridging methoxide ligands; however, this dimer is a *cis*-dioxo rather than mono-oxo molybdenum compound like compounds 2 and 3, which is also unique in having an additional bridging THF ligand.^47^ Other similar dimeric examples often contain ammonium,^48^ phosphonium,^49^ alkali,^49^ or alkaline earth^50,51^ cations, co-crystallized solvents like water,^51,52^ ethanol,^53^ methanol,^54^ toluene^55^ or benzene.^56^ Coordinating solvents like water,^51^ ethanol,^53^ or pyridine^48^ along with bridging oxo,^48^ alkoxide,^54,56^ phosphate,^57^ hydroxide,^58^ oxalato,^48^ citrato,^59^ or dioxomolybdenum^60^ groups either between the Mo dimer atoms or bridging pairs of dimers has also observed. This is like the crystal structure observed for the polymeric compound 3, C5b (Fig. 3). Understanding the structure of small molybdenum clusters that are candidates for electrodeposition is crucial, and this literature and work highlights many unique coordination states surrounding molybdenum with respect to small multidentate oxygen-based ligands. The chemical structure of any given metal precursor could vary in number of metal atoms, oxidation states, geometry, ligands, and more – all are crucial aspects of a reagent for understanding and elucidating valid chemical mechanisms for the electrodeposition processes of these critical materials such as MoS_2_.

The polymeric structure of compound 3 (C5a) exhibits two unique bridging interactions with ethylene glycoxide ligands, bridging the molybdenum dimer atoms either parallel O11–(Mo1–Mo2) or perpendicular O12–(Mo3–Mo4) to the molybdenum/oxo double bond (Fig. 3). An oxalic acid bimetallic Mo-coordination complexes was observed to undergo polymerization through an oxalic acid bridging ligand; however, the presence of bridging oxo (O^2−^) ligands may prevent further polymerization as the oxo bridging ligand tends to directly bridge the molybdenum atoms with no spacer like the C_2_ backbone of ethylene glycoxides or oxalates resulting in increasingly larger clusters, rather than polymer comprised of the dimer units.^48,61,62^ An additional example of an oxalate polymer exists with R–MoO_2_–R bridging units (R = Mo dimer, (MoO(oxalato)(μ-oxo))_2_) and cations; however, for this polymeric structure, the bridging MoO_2_ unit is not directly involved in bridging the two Mo atoms in each dimer that was also observed for a phosphate bridged dimer.^57,60^ This is not like what is observed for compound 3 with ligands involved in the polymerization coordinating through both molybdenum atoms of the dimer (Fig. 3) rather than from one molybdenum atom to the second in another dimer molecule.

**Fig. 4:**
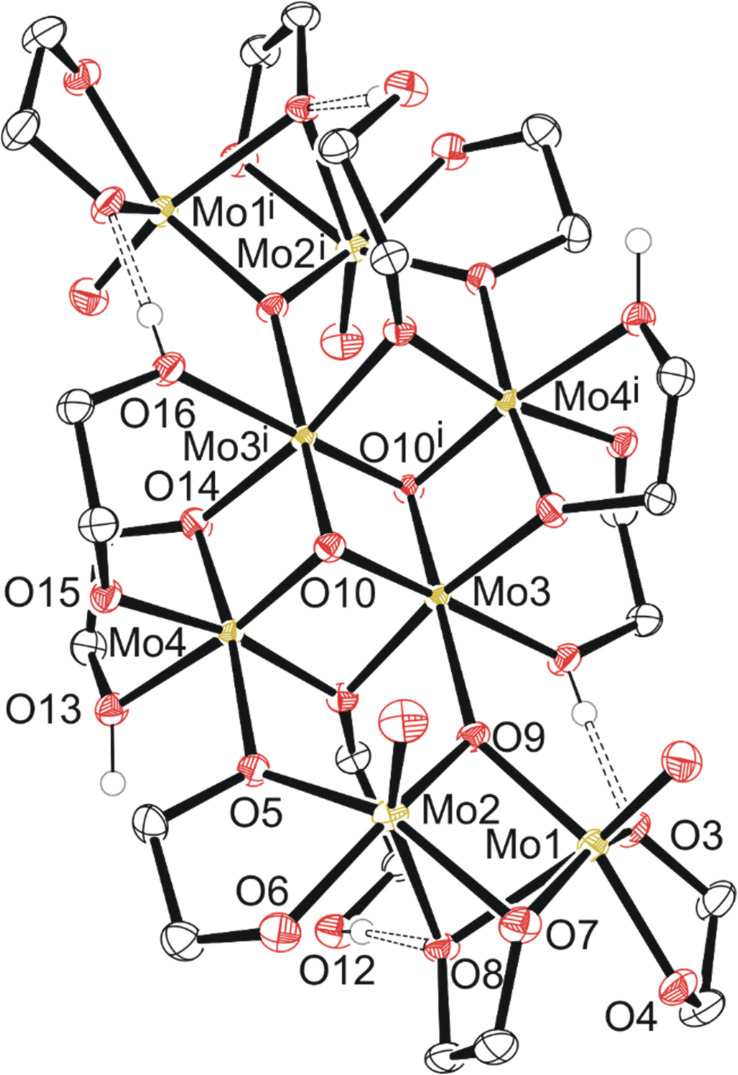
Molecular structure of compound 4 with anisotropic displacement ellipsoids projected at the 50% probability level. Methylene CH_2_ hydrogen atoms have been omitted for clarity.

Compound 4, C6a (Fig. 4) exists as a cluster showing the octameric eight molybdenum atoms consisting of a distinctly different core of four with two Mo(v) dimers like 2 and 3 on each side of the core. Each dimer is equivalent across a symmetry element, and the dimers are held together by a μ-oxo ligand and one of the two oxygen atoms of the bidentate ethylene glycoxide that attaches the dimer to the tetrameric central core. Each dimer also has a bridging ethylene glycoxide ligand bound directly to both molybdenum atoms. The central tetrameric core with Mo 3/4 has two distinct molybdenum environments as well, forming a diamond shape. Mo3 and symmetry equivalent Mo3^i^ exists with three μ_3_-oxo ligands, a neutral monodentate ethylene glycol hydroxy ligand, and two μ-ethylene glycoxide ligands.

**Scheme 2:**
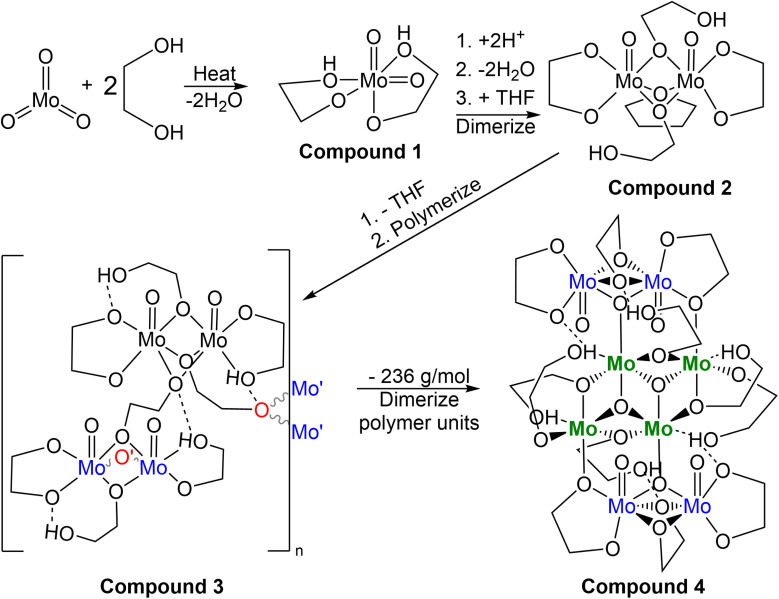
Hypothetical reactions relating observed compounds.

From Table S3,† the masses of 1–4 are ∼250, 538, 932, and 1766 g mol^−1^, respectively. Assuming 1 turns into 2, then 3, and finally 4, one pathway (Scheme 2) regarding conservation of mass would be for two molecules of 1 (2 × 250 g mol^−1^) to be protonated (+2 × 1 g mol^−1^) followed by loss of 2H_2_O (−2 × 18 g mol^−1^) while gaining the THF unit (+72 g mol^−1^) to equal 538 g mol^−1^ as 2. Subsequent loss of THF (466 g mol^−1^) and polymerization through ethylene glycol fragments affords 3 with discreet dimers with a mass equal to twice that of 2, ∼932 g mol^−1^. The final conversion from two molecules of 3 into 4 requires a shift from 932 + 932 = 1864 g mol^−1^ (2 × 3) into 1628 g mol^−1^ (excluding 2× ethylene glycol solvent molecules of 4). This is a net loss of 236 g mol^−1^ comprised of 8 oxygen, 8 carbon, and 18 hydrogen atoms. The central cluster contains 2 oxo groups to 4 molybdenum, implying loss of 4 oxygen atoms through loss of oxo groups between a pair of dimers is necessary for creation of the central tetrameric core of 4.

This conversion could be mediated by protonation from hydroxy groups from solvent ethylene glycol, entirely hydrogen-bound ethylene glycol molecules such as that observed in the crystal structure of 4, or through the singly deprotonated ethylene glycol mono- or bidentate ligand (2-hydroxyethyl-1-oxo) commonly observed hydrogen bonding intramolecularly such as in the crystal structures of 1 and 2 monodentate ligand (Table S4†). The complexity of this final conversion if this is the route towards formation of 4, is too great for any degree of certainty for its formation.

Based on the formula of the Mo1–Mo2 dimer, a doubly bridging bidentate ethylene glycoxide ligand is possible, meaning singly bridging mono-deprotonated ethylene glycol ligands observed in crystal structures of 2–4 could participate in proton transfer. This could result in a variety of observed intramolecular motifs such as the bidentate non-bridging ethylene glycoxide oxygen atoms and oxo groups.

The protic nature of the solvent and ligands allows for the coupling of molybdenum clusters *via* protonation of the bridging glycoxide, dissociation or rearrangement of the newly formed hydroxy group, insertion of the newly formed ethoxide ligand, with the intermediate cationic dimer. The presence of a bridging oxo group between molybdenum atoms of the dimer and the central tetramer of compound 4 could be the result of hydrolysis of the C–O bond of a bridging ethylene glycoxide unit, forming a bridging hydroxy group OH. If this bridging hydroxy group were to encounter a Mo–OH, loss of water and formation of the observed triply bridging oxo groups partially bonding the central tetramer with the outer dimers. Much of this is speculative; however, possible steps are presented by which interconversion from the monomeric MoO_2_(OC_2_H_4_OH) to larger clusters observed in this work may occur. The opposite may be true as well, with incomplete dissolution resulting in the clusters undergoing reduction and extensive hydrolysis, loss of water, and formation of ethylene glycol units. Additional crystallographic information is included in the supplemental information (Tables S3–S5†).

### Electrochemistry

Electrochemical studies began with analysis of the relevant precursors (1,4-butanedithiol, ethylene glycol, B3a, and B6a) using a supporting electrolyte solution comprised of 0.1 M PP13-TFSI in THF. Various electrochemical techniques were employed for characterization and electrodeposition including cyclic voltammetry (CV), chronoamperometry (CA), and bulk electrolysis (BE) in either 0.1 M PP13-TFSI in THF or in the pure PP13-TFSI ionic liquid (heated to ∼100 °C to facilitate mixing).

#### PP13-TFSI in THF on glassy carbon and platinum working electrodes

The supporting electrolyte used for electrochemical studies was 0.1 M PP13-TFSI in THF. Rather than using an alternate electrolyte solution, PP13-TFSI was used as the electrolyte in case it is necessary for the reductive conversion of the Mo(vi) precursors to Mo(iv) disulfide. This electrolyte and solvent combination possesses an electrochemical window of −1.25 to 0.5 V *vs.* Ag/Ag^+^ conducted within a 50–100 ppm O_2_ glovebox (Fig. S13†) with maximum current of ∼2 μA. It should be noted as well that while reduction current grows exponentially during the sweep from −2.5 V to −3.0 V, current remainsbelow ∼25 μA. The potentiostat, laptop computer, and the electrochemical cell and cables were all present within the glovebox and manipulations were carried out entirely within the glovebox. For these preliminary studies, PP13-TFSI in THF showed potential electrochemical stability. This is a key advantage of non-aqueous electrochemistry that allows for very low voltage cathodic reduction, which appears important for reduction of molybdenum-based precursors directly into crystalline MoS_2_ rather than amorphous films of MoS_*x*_.

**Fig. 5:**
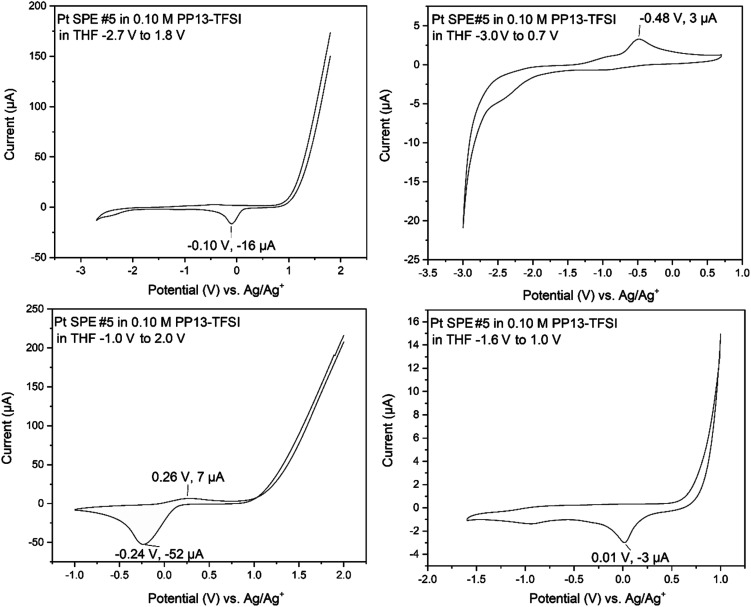
Electrochemical window elucidation for the 0.10 M PP13-TFSI in THF solution used for later electrochemical studies. Order of collection on the same electrode in solution follows top left (100 mV s^−1^), top right (50 mV s^−1^), bottom left (50 mV s^−1^), then bottom right (50 mV s^−1^).

Initial exploration of the full electrochemical window of PP13-TFSI in THF on Pt (Fig. 5) highlighted oxidation above +0.5 V with subsequent reduction occurring between −0.25 V and 0 V on the cathodic scan. The first window scanned was −2.7 V to +1.8 V, resulting in significant oxidation occurring above +1.0 V and a reduction event at −0.1 V with very little further activity. When the window was reduced to −1.0 V to +2.0 V, a significant oxidation above +1.0 V was observed with a broad reduction beginning at ∼+0.2 V, peaking at −0.24 V. Changing the window to −1.6 V to +1.0 V results in a similar oxidation behavior occurring at ∼+0.5 V. The cathodic region for this electrolyte is large, with little current registered to nearly −2.7 V. When the window of −3.0 V to +0.7 V was explored, reduction occurring from −2.0 V to −3.0 V resulted in an oxidation event on the following anodic scan at −0.48 V. This work indicates that this ionic liquid is effective for depositions at the negative voltages proposed by Murugesan *et al.*;^1^ however, positive voltages above +0.5 V result in oxidation and should thus be avoided.

On a glassy carbon WE with a platinum wire CE and an Ag/Ag^+^ RE (0.1 M PP13-TFSI in THF), the electrochemical window (Fig. S14†) was stable between −1.25 V and +0.50 V. Reduction was observed beginning at ∼−1.6 V; however, a relatively stable electrochemical window between −2.0 V and +0.5 V was also observed. Altering the electrochemical window to −2.7 V to +0.5 V resulted in a broad reduction with two main features at roughly −2.2 and −2.7 V, respectively, causing a broad irreversible oxidation signal spanning −1.75 to −0.5 V with a peak of unknown origin at −0.9 V. The current observed in the CV of the electrolyte solution between −3.0 to 0.7 V is significantly less than is observed when analytes are included within the solution showing support that it is an ideal solution for −2.7 V necessary to produce MoS_2_ according to Murugesan *et al.*^1^ Following the characterization of the electrochemical window of the 0.10 M PP13-TFSI in THF, 1.5 mL of this solution had 0.05 mL of various reagents present during MoS_2_ electrodeposition attempts as detailed later. The reagents studied include ethylene glycol, 1,4-butanedithiol, a mixture of ethylene glycol and 1,4-butanedithiol, and molybdenum-based precursors (B3a, B6a) respectively.

#### 1,4-Butanedithiol in 0.1 M PP13-TFSI in THF

To better understand the electrochemical behavior of 1,4-butanedithiol and other reagents under these electrochemical conditions, some control experiments were performed. Cycling between −2.7 V and +1.0 V using Pt SPE #1 at 50 mV s^−1^ in 1.5 mL of 0.1 M PP13-TFSI in THF and 0.05 mL 1,4-butanedithiol resulted in clearly defined oxidation and reduction peaks with significant separation of peak voltages of ∼2 V (Fig. S15†).

Adsorption onto the platinum surface upon cathodic reduction may occur below roughly −1.2 V, registering current that rapidly dissipates with a maximum current at roughly −2.5 V, decreasing towards −2.7 V. Beginning the anodic scan from −2.7 V to +1.0 V also occurs with a sharp increase in current. This indicates adsorption of the material to the electrode surface, and desorption is observed above roughly −0.8 V. Support is provided by SEM-EDS analysis of the electrode surface following the desorption process, where the surface is free of sulfur (Fig. S16†), while it otherwise shows sulfur and carbon by SEM-EDS if the CV finished at −2.7 V. This characterization indicates that the 1,4-butanedithiol is electrochemically active within PP13-TFSI in THF and is undergoing an electron transfer process below −1.2 V, and above −0.8 V upon the following anodic scan.

#### Ethylene glycol in 0.1 M PP13-TFSI in THF

The electrochemical behavior of ethylene glycol was studied due to a possible residual presence of ethylene glycol within the molybdenum-based precursors. To 1.5 mL PP13-TFSI in THF, 0.05 mL ethylene glycol was added. Cycling between −2.7 V and +1.0 V at 50 mV s^−1^ resulted in the appearance of a significant resistive component in the CV beginning at roughly −1.0 V (Fig. S17†). Appearance of this resistive component in the CV indicates that there is resistance to current flow which may be due to the increased viscosity of the electrolyte after addition of ethylene glycol, which may impede electron flow. Two events occur during the anodic scan at roughly −0.9 V and −0.3 V that may correspond to adsorption and/or phase reorientation of the ethylene glycol from the WE surface.

#### Ethylene glycol and 1,4-butanedithiol in 0.1 M PP13-TFSI in THF

**Fig. 6:**
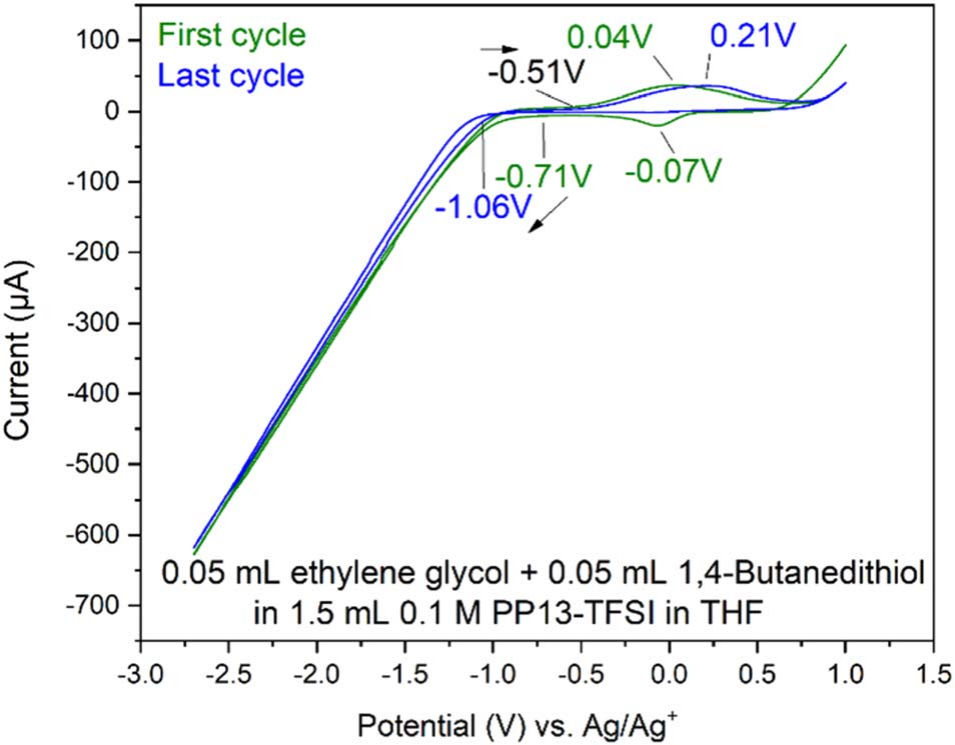
CV of a mix of 0.05 mL ethylene glycol and 0.05 mL 1,4-butanedithiol added to 1.5 mL 0.1 M PP13-TFSI in THF with 7 cycles between −2.7 V and +1.0 V at 50 mV s^−1^ on Pt SPE #2.

Following the previous experiments with 1,4-butanedithiol and ethylene glycol, 0.05 mL of both species were added to 1.5 mL PP13-TFSI in THF, and cyclic voltammetry was performed (Fig. 6). This combination was explored to see if electrochemical behaviour would differ from the two pure compounds. An increase in current upon cathodic reduction is observed around −1.0 V. Between −1.0 V and −0.5 V nothing occurs for the anodic scan, and this is where the first of the two ethylene glycol peaks were observed (−0.86 V). Addition of ethylene glycol alters the redox chemistry of the pure 1,4-butanedithiol as observed in Fig. S15.†

#### Molybdenum-based precursors in 0.1 M PP13-TFSI in THF

**Fig. 7:**
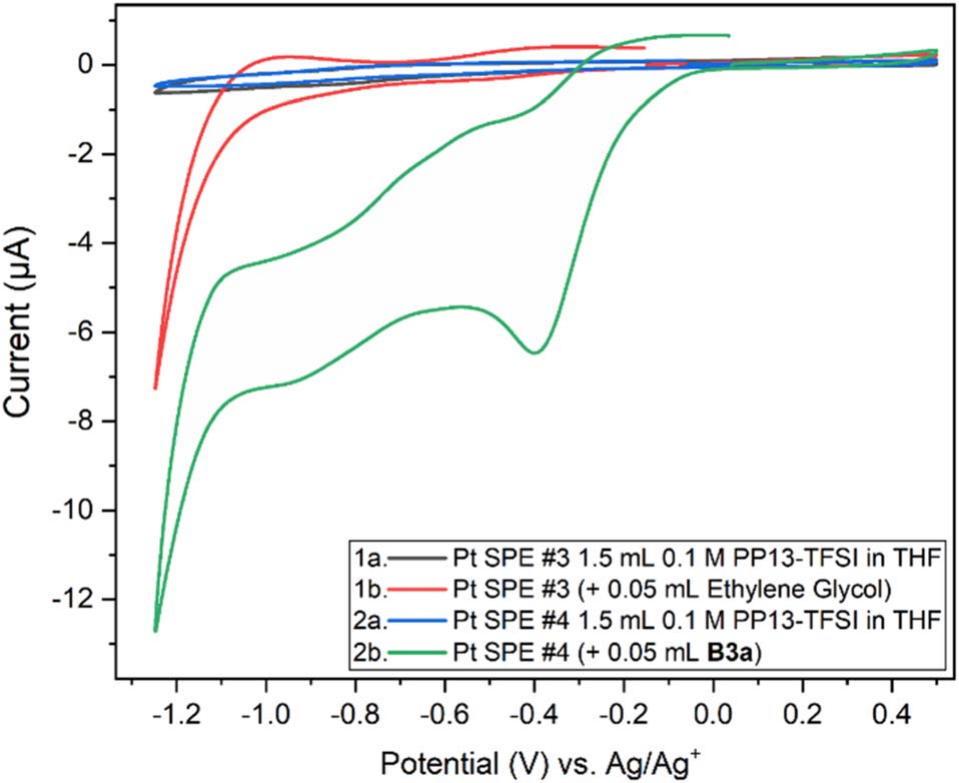
Cyclic voltammogram from OCP to +0.5 V, then to −1.25 V and back to the starting voltage for solutions of 1.5 mL 0.1 M PP13-TFSI in THF with the addition of 0.05 mL ethylene glycol (left) or B3a (right) at 10 mV s^−1^.

The molybdenum-based precursors contained residual ethylene glycol, and as such a comparison of the electrochemical behaviour between the ethylene glycol used as starting material, the gold B3a, and the brown B6a samples were done. It is important to note that as we are fully unaware of the solution-state identity of these molybdenum compounds, we refrain from speculation into the nature of the oxidation and reduction events for the incompletely understood molybdenum precursors. Comparison of 0.05 mL ethylene glycol and 0.05 mL B3a within the 1.5 mL PP13-TFSI in THF solution and their individual open circuit potential (OCP) resulted in significant differences between pure ethylene glycol and the molybdenum saturated golden viscous liquid B3a (Fig. 7).

**Fig. 8:**
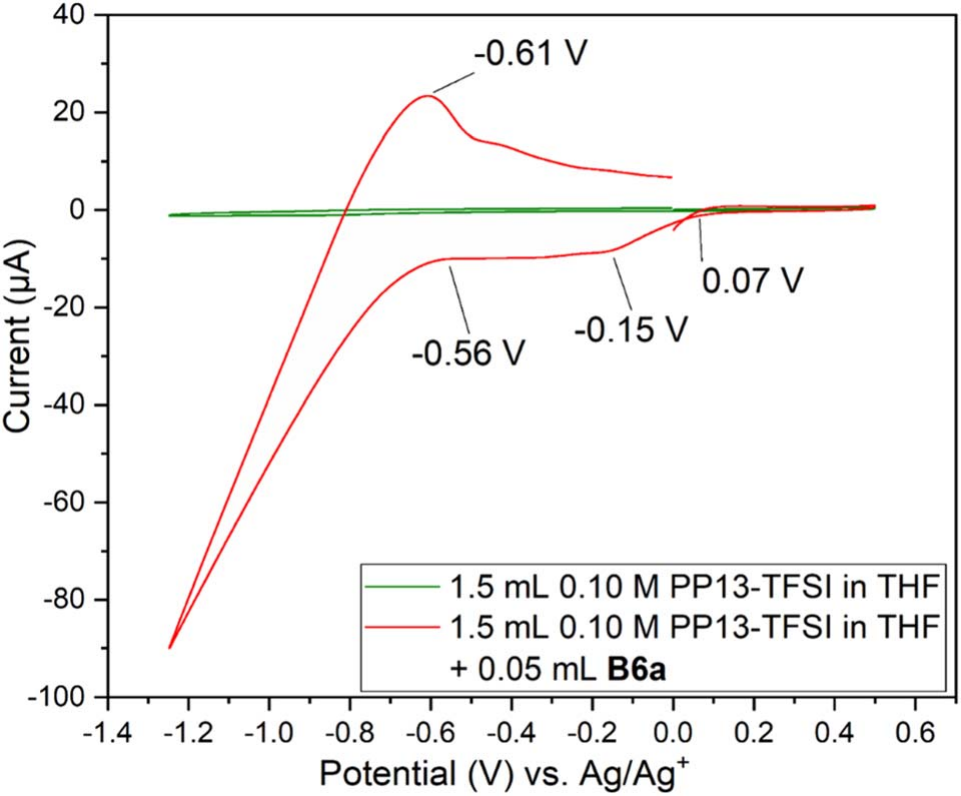
Cyclic voltammogram cycle from OCP to +0.5 V, then to −1.25 V and back to the starting voltage for solutions of 1.5 mL 0.1 M PP13-TFSI in THF, followed by addition of 0.05 mL B6a at 50 mV s^−1^ on Pt SPE #1.

Two Pt SPE (#3 and #4) with similar electrochemically active surface areas (Fig. S12,† total current (and thus adsorbed hydrogen and relevant electrochemical surface area) are similar in 0.5 M H_2_SO_4_)^64^ were used for this study. Initial cycling from −1.25 V to +0.5 V in the blank electrolyte solution resulted in a clean voltammogram (Fig. 7). Subsequent addition of each species and repeat scanning at 10 mV s^−1^ from OCP to +0.5 V, down to −1.25 V and back to the original OCP voltage gave rise to a significant difference. The choice to scan from OCP was to avoid initial oxidation or reduction which could occur otherwise. Ethylene glycol alone maintained the same double layer current as the blank solution until roughly −0.7 V during the cathodic scan, with reduction beginning around −1.0 V. The B3a in contrast experienced an increase in the current beginning at about 0 V, with a peak at −0.4 V. An additional increase in current is observed beginning at −0.7 V with another increase below −1.1 V. The current registered with B3a is greater than that of ethylene glycol; however, the initial blank solution for each system was similar. Upon anodic scanning from −1.25 to +1.0 V, B3a gave had an increase in current between −1.25 and −1.1 V, with a very broad increase between −0.8 and −0.4 V. Though a different scan rate was used when analyzing the brown viscous liquid B6a, it is clear that B3a and B6a have very different electrochemical features upon cycling once from −1.25 V to +0.5 V (Fig. 8). The golden B3a displays reduction events at ∼−0.4 V and ∼−0.6 V, beginning with a resistive increase in current at ∼−1.0 V (Fig. 7). In contrast, the brown viscous liquid B6a has no reductive event at ∼−0.4 V which is the major distinguishing feature of the CV containing B3a. Unlike ethylene glycol or B3a, B6a is observed to begin a cathodic reduction beginning at ∼−0.6 V which does match a reduction event of B3a; however, there exists no analogous plateau in current like that observed for B3a prior to the resistive increase in current below −1.0 V observed for both pure ethylene glycol and B3a. A unique observation within the B6a sample is a broad oxidation event occurring at ∼−0.6 V during the anodic scan.

Analysis by SEM-EDS indicated the presence of molybdenum, confirming that the brown viscous liquid B6a does deposit onto the platinum working electrode following a single scan from 0 V to +0.5 V to −1.25 V and back to 0 V (Fig. S20†).

These ethylene glycol/glycoxide molybdenum compounds are amphoteric (potentially acidic and basic) and as such there exist many possible mechanistic routes to the simpler binary compound MoS_2_. This substantial chemical conversion from the suspected initially formed precursor candidate MoO_2_(C_2_H_5_O_2_) to MoS_2_ is likely to involve a combination of reductive elimination, protonation, deprotonation, loss of ethylene glycol/glycoxide ligands, loss of water (through –oxo group double protonation), and eventual coordination of thiol/thiolate ligands with subsequent breaking of S–C bonds, losing the 1,4-dibutyl fragment.

#### Attempts to electrodeposit MoS_2_ using literature conditions

Following successful synthesis of a MoO_3_/ethylene glycol reaction mixture which was brown B6a, deposition was conducted following the procedure by Murugesan *et al.*^1^ The electrochemical conditions that were successful for their reported deposition involved a mixture of 1.5 mL pure PP13-TFSI ionic liquid as both solvent and electrolyte with addition of 100 μL of both 1,4-butanedithiol and the brown viscous liquid molybdenum-precursor. To this mixture at 100 °C, a chronoamperometric deposition at −2.7 V for 600 seconds (Fig. S18†) was conducted and the electrodes were rinsed with acetone and dried leaving the electrode with minor sulfur containing material lacking the desired MoS_2_ nanoflower morphology and clear crystalline structure (Fig. S19†). In the original work, a 1 × 1 cm piece of glassy carbon was used as the working electrode, mounted on a rotating disc electrode with carbon tape (a potential source of our attempts being unsuccessful). Platinum was used for both counter and quasi-reference electrodes. Different to this group, Ag/Ag^+^ was used as a reference electrode in non-aqueous conditions, and the glassy carbon working electrode was not sonicated following 5 μm alumina polishing.

The ionic liquid was mixed with the molybdenum-precursor prior to heating in a low volume 3 electrode cell within a sand bath on a hotplate. Homogeneity of the mixture was not observed until ∼50 °C. Addition of 1,4-butanedithiol was done at ∼75 °C, which resulted in an immediate dark black precipitate forming which was not mentioned in the work by Murugesan *et al.*^1^ Prior to immersion of the electrodes, the majority of the solid was scraped from the solution surface and removed; however, this may be what resulted in crystalline MoS_2_ shown by Murugesan *et al.* The electrode was immersed at ∼80 °C, and upon reaching 100 °C, chronoamperometric deposition at −2.7 V for 600 seconds was conducted.

Following this electrodeposition, the electrode was rinsed with THF and stored in a desiccator. SEM analysis of the electrode revealed abundant debris, including fragmented material with sharp edges and sparse globular material. Analysis by SEM-EDS revealed the sharp material contains aluminum, indicating residual alumina from polishing. Analysis of the globular material revealed a signal for sulfur; however, in both instances signal intensity was mostly carbon (Fig. S19†). The crystalline MoS_2_ nanoflowers reported by Murugesan *et al.* could not be reproduced. Because of the substantial difficultly in combining these thick liquids, the desire to avoid heating 1,4-butanedithiol near its boiling point, the formation of the unexpected precipitate, and the desire to produce MoS_2_ under more ideal working conditions while using minimal costly ionic liquid, experiments were mostly conducted using a solution approach at room temperature.

#### Attempts to electrodeposit MoS_2_ by room temperature non-aqueous solutions

Use of the 0.1 M PP13-TFSI in THF supporting electrolyte at room temperature was explored to attempt MoS_2_ electrodeposition using a ratio of 1.5 mL 0.1 M PP13-TFSI in THF to 0.05 mL 1,4-butanedithiol and 0.05 mL of B3a and B6a. In general, these attempts would result in no significant deposit when observed by SEM, while SEM-EDS mostly showed pure platinum, or sulfur, carbon, and oxygen. Amorphous deposits were achieved in a few instances with either sulfur or molybdenum present; however, the overlap of their signals during SEM-EDS analysis resulted in an inability to discern the composition (Fig. S20†). The issues associated with the unexpected precipitate material that forms when mixing the molybdenum and sulfur precursors in pure PP13-TFSI was found to be avoided by mixing the precursors in the presence of THF. When solutions were prepared and used right away, no precipitate formed and the solution colour changed to red like in the pure ionic liquid. Since the brown viscous liquid B6a was not available for early studies, the gold viscous liquid B3a was used for the majority of the alternative deposition attempts. The use of this supporting electrolyte solution with screen-printed platinum electrodes in a compact voltammetry low volume cell was used for most studies, although this electrochemical cell could not be heated as per manufacturer recommendation. Use of the screen-printed platinum electrodes allowed for minimal solvent usage and access to multiple electrodes of similar fabrication to both explore and repeat experiments. Use of PP13-TFSI in THF as a supporting electrolyte also allowed for increased conservation of this expensive material.

When mixing the gold B3a (0.05 mL) with 0.1 M PP13-TFSI (1.5 mL), a pale-yellow solution colour was observed due to dilution. As a compatibility test, addition of 0.05 mL 1,4-butanedithiol to this solution caused a colour change to orange, then to red over the course of 30 minutes, with a red precipitate settling out over the course of a week. The clarified red liquid was then used for deposition onto the Pt WE *via* first applying −2.7 V for 600 seconds, followed by a single scan from −0.75 V to −2.7 V at a scan rate of 50 mV s^−1^ (Fig. S21†).

**Fig. 9:**
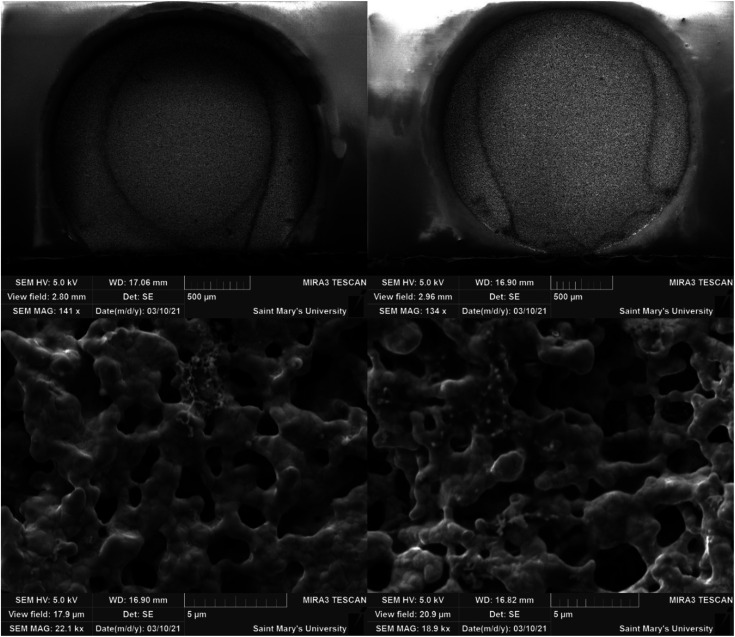
SEM secondary electron images of electrodes (left = Pt SPE #3, right = Pt SPE #4) showing amorphous, non-homogenous deposit following a series of bulk electrolysis (−2.7 V for 600 seconds) following by a single scan from −0.75 V to −2.7 V at 50 mV s^−1^.

**Fig. 10:**
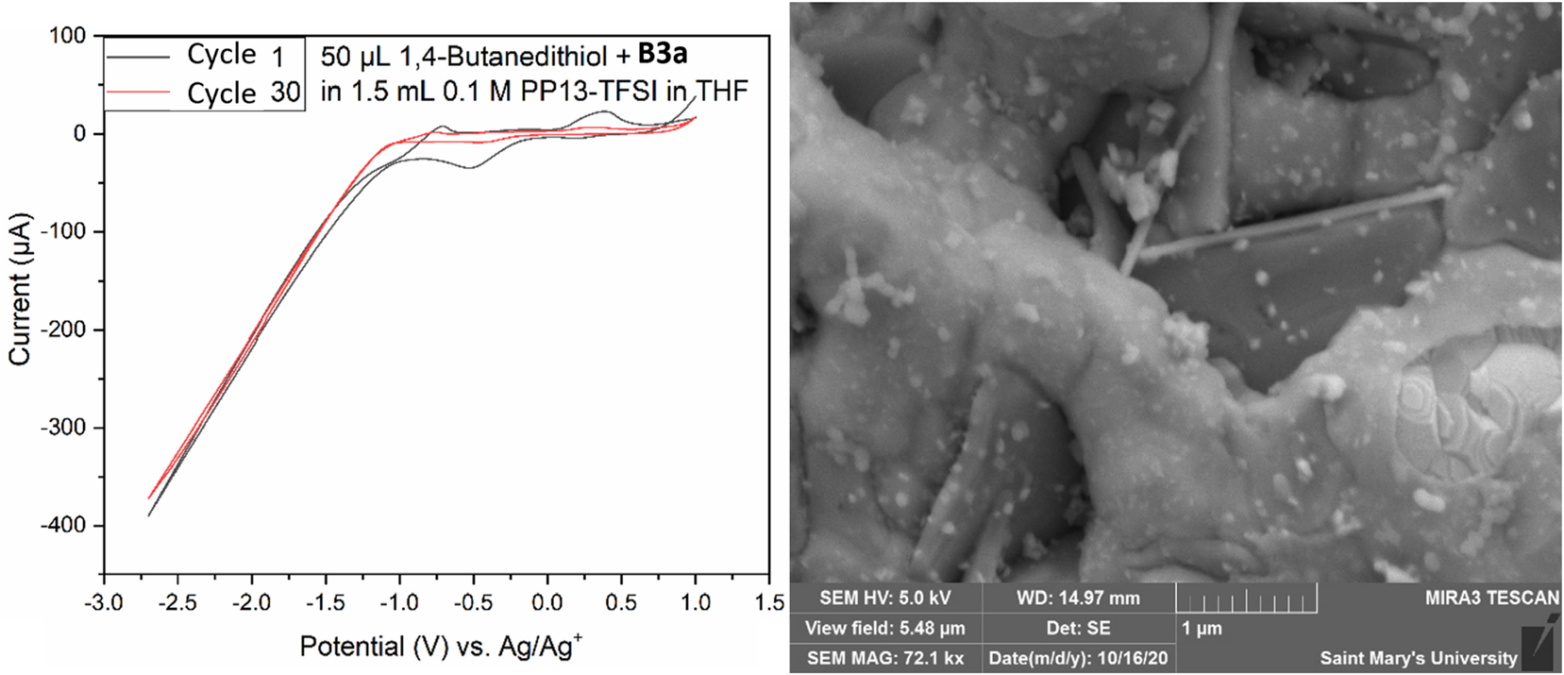
Cyclic voltammogram (left) of the attempted electrodeposition of MoS_2_ from a mix of 1.5 mL 0.1 M PP13-TFSI in THF, 0.05 mL 1,4-butanedithiol, and 0.05 mL B3a. The first and 30th cycle are shown from −1.0 V to −2.7 V with a scan rate of 50 mV s^−1^. SEM SE image of the surface of the electrode following deposition of a nanoscale material. Long bladed crystals are a mix of Al/Zn/O from electrode fabrication.

Both electrodes had a deposit as evidenced by SEM analysis (Fig. 9); however, the morphology presented as a non-homogenous ring around the electrode, existing as a globular amorphous deposit upon closer examination. Two solutions were prepared containing 50 μL 1,4-butanedithiol, B3a, and either 1.5 mL THF, or 1.5 mL 0.1 M PP13-TFSI in THF. The THF solution appeared to have an orange suspended solid, and the 0.1 M PP13-TFSI in THF initially turned orange, changing to red with a red precipitate settling out after one week. The THF solution without ionic liquid remained as a turbid orange suspension. The reddish orange solution from the 0.1 M PP13-TFSI in THF was decanted and used as electrochemical electrolyte. SEM-EDS analysis revealed the presence of sulfur and platinum, with other metal signals (Fe, Mg, Co) potentially present as artifacts or as contaminants. An additional deposition attempt was conducted in a solution of 1.5 mL 0.1 M PP13-TFSI in THF that had 0.05 mL B3a added to it, followed by 0.05 mL 1,4-butanedithiol in a low volume cell with a screen-printed platinum electrode (Pt SPE #5). CV from +1.0 V to −2.7 V for 30 cycles at a scan rate of 50 mV s^−1^ was run to attempt deposition. The voltammogram had a reduction peak at +0.5 V followed by a significant increase in reductive current from −1.0 V to −2.7 V. The diagonal component to the voltammogram at these negative potentials indicates a resistive component to the circuit which may be due to the viscosity of residual ethylene glycol at the electrode surface. There is an oxidation peak at ∼−0.7 V and another at ∼+0.4 V. After 30 cycles the current between −1.0 V and +1.0 V increases significantly, and the redox features are no longer observed (Fig. 10). This decrease in current may indicate the formation of a stable and thick insulating layer on the Pt surface which does not allow for current to flow as readily.

This work was not conclusive regarding the state of each deposit; however, various unique morphologies were observed by SEM analysis following electrodeposition by chronoamperometric and cyclic voltametric methods within 0.1 M PP13-TFSI in THF as a supporting electrolyte solution and 1,4-butanedithiol as a sulfur precursor and concentrated MoO_3_ and ethylene glycol reaction solutions. SEM-EDS analysis indicated the presence of sulfur and molybdenum.

These experiments indicate that PP13-TFSI in THF as a supporting electrolyte has a wide electrochemical window. A solution of 0.1 M PP13-TFSI in THF does display a resistive component from −1.25 V to +0.5 V, indicating higher concentrations of electrolyte may perform as a better non-aqueous electrodeposition matrix. Electrochemical analysis of ethylene glycol indicated that its presence in solution at the concentrations studied may be problematic, introducing an apparent resistive component to the electrochemical system which impedes current flow. There is difficulty associated with complete removal of ethylene glycol (b.p. 197 °C) from the molybdenum-precursor proposed by Murugesan *et al.* without potentially changing its composition considerably. Issues of irreversible decomposition processes including thermally induced changes (too much heat = black solid), solvent mediated chemical alterations (water reacts with the studied molybdenum-precursors to form a blue compound thought to consist of large poly-oxomolybdate clusters, and THF coordinates to Mo dimers *via* μ-bridging of the metal centers), or a variety of clustering processes (dimerization, polymerization, tetra-/octamerization). SEM-EDS and CV measurements indicate that material from electrodeposition attempts containing either sulfur or molybdenum has been deposited; however, the current lack of reproducibility between batches of the key molybdenum precursor prevented further characterization of these undesirable non-homogenous amorphous films.

## Conclusion

A literature procedure for producing MoS_2_*via* electrodeposition in the ionic liquid PP13-TFSI at 100 °C from 1,4-butanedithiol as the source of sulfur and crude reaction mixtures of MoO_3_ with ethylene glycol as the source of molybdenum was explored. Replication of this procedure using the reported −2.7 V, 300 seconds chronoamperometric electrodeposition conditions using the sixth molybdenum-precursor synthesized, which was most akin to the required “brown viscous liquid” was unsuccessful. During the attempt to replicate the published work, four crystalline materials were isolated, giving insight into the reaction's complexity. This represents monomeric (1), dimeric (2), polymeric (3), and a larger cluster containing eight molybdenum atoms (4). In addition, electrochemistry using 0.1 M PP13-TFSI in THF as a non-aqueous, polar aprotic electrolyte solution was conducted on both platinum and glassy carbon working electrodes. Electrochemical analysis of ethylene glycol, 1,4-butanedithiol, a mix of the two, and various liquid molybdenum-precursor samples was performed. Various attempts were made to electrodeposit MoS_2_ within this electrolyte solution, and SEM-EDS analysis indicates non-homogenous coverage of amorphous deposits containing sulfur and molybdenum from room temperature solutions, although overlap of their X-ray signals gave inconclusive results and a ratio could not be determined. Indications of the existence of [PP13]_2_[Mo_6_O_19_] (5) as an oxidation biproduct on quenching the precursor solution with 1 : 1 v/v 5% sodium hypochlorite was observed. The variety of diversity within the MoO_3_ ethylene glycol reaction warrants further investigation into the active component that allowed for direct electrodeposition of MoS_2_ as reported by Murugesan *et al.* One or more of these compounds may be responsible for the successful direct electrodeposition of molybdenum disulfide by Murugesan *et al.*, but current knowledge is very limited and further work would be needed to find a reliable mechanism for MoS_2_ formation, in addition to studying the previously reported monometallic (1) as a precursor, alongside synthesizing the lower oxidation state molybdenum cluster compounds (2–4) to also test as precursors.

This work ended up far more fundamental than intended resulting in incompletely characterized, yet conclusively identified molybdenum compounds in the as-yet incompletely understood brown viscous liquid. This work represents a multifaceted study into aspects surrounding the first report to our knowledge of direct electrodeposition of crystalline MoS_2_. The limits identified by our synthetic and electrochemical studies indicate that understanding the chemical identity of “active” intermediates is key within complex redox processes. For example, electrodeposition of binary compounds like MoS_2_ would take a number of steps from more complex precursors like those we have found in our attempts to create the brown viscous oil molybdenum precursor reported by Murugesan *et al.*^1^

## Author contributions

Data curation, formal analysis, methodology, project administration, writing (review/editing), and conceptualization was contributed by all authors. Christa and Jason each contributed to funding acquisition, resources, and supervision. Tanner performed the investigation, validation, visualization, and original draft writing within his MSc thesis work (2021).^65^

## Conflicts of interest

No conflict of interest exists with any authors and this work.

## Supplementary Material

RA-013-D3RA04605B-s001

RA-013-D3RA04605B-s002
