# Correction: Electrochemical and X-ray structural evidence of multiple molybdenum precursor candidates from a reported non-aqueous electrodeposition of molybdenum disulfide

**DOI:** 10.1039/d4ra90007c

**Published:** 2024-01-25

**Authors:** Tanner George, Christa L. Brosseau, Jason D. Masuda

**Affiliations:** a Department of Chemistry, Saint Mary’s University Halifax Nova Scotia B3H 3C3 Canada Tanner.George@smu.ca

## Abstract

Correction for ‘Electrochemical and X-ray structural evidence of multiple molybdenum precursor candidates from a reported non-aqueous electrodeposition of molybdenum disulfide’ by Tanner George *et al.*, *RSC Adv.*, 2023, **13**, 32199–32216. DOI: https://doi.org/10.1039/d3ra04605b

The authors regret that incorrect references were reported in the original article. On the second paragraph of page 32200 of the original article, the sentence beginning with “This gave insight into an earlier literature synthesis…” should be changed to “This gave insight into an earlier literature synthesis^46^ and crystal structure^34^ related to this preparation which heats the crude MoO_3_ + ethylene glycol mixture to 150 °C to produce MoO_2_(OC_2_H_4_OH)_2_.”

Ref. 34 and 46 are listed here as ref. 1 and 2, respectively.

The Royal Society of Chemistry apologises for these errors and any consequent inconvenience to authors and readers.

## Supplementary Material
