# Supplementary material for: Electrochemical and X-ray structural evidence of multiple molybdenum precursor candidates from a reported non-aqueous electrodeposition of molybdenum disulfide
Source: RSC Adv. 2023 Nov 1;13(46):32199–216. doi: 10.1039/d3ra04605b (PMC10619629; doi:10.1039/d3ra04605b)
Supplement: RA-013-D3RA04605B-s001 [file RA-013-D3RA04605B-s001.pdf]

Electronic Supplementary Material (ESI) for RSC Advances.
This journal is © The Royal Society of Chemistry 2023

Supporting Information

Electrochemical and X-Ray Structural Evidence of Multiple Molybdenum Precursor Candidates from a Previously Reported Non-Aqueous Electrodeposition of Molybdenum Disulfide

Tanner A. George,* Jason D. Masuda,* Christa Brosseau*

Department of Chemistry, Saint Mary's University, Halifax, Nova Scotia, Canada B3H 3C3.

Contents

Ionic Liquid 3
Synthesis of PP13-Br cation precursor 3
First Synthesis of PP13-TFSI 3
Second Synthesis of PP13-TFSI 4
Third Synthesis of PP13-TFSI 4
Fourth Synthesis of PP13-TFSI 4
NMR Spectroscopy of Ionic Liquid, Precursors, and 1,4-Butanedithiol 5
Molybdenum Precursor 9
Synthesis Details 9
First synthesis of the Molybdenum Precursor – A1 9
Second Synthesis of the Molybdenum Precursor – A2 10
Fourth Synthesis of the Molybdenum Precursor – A4 10
Seventh Synthesis of the Molybdenum Precursor – A7 11
Eighth Synthesis of the Molybdenum Precursor – A8 11
Crystallography 13
Ratio of Molybdenum and Oxygen 17
Electrochemistry, Electrodeposition, and SEM Analysis 18

Ionic Liquid

To prepare the ionic liquid solvent PP13-TFSI, Li-TFSI was used as the anion ($TFSI^-$) precursor while the cation precursor PP13-Br was prepared synthetically from N-methyl piperidine and 1-bromopropane via a modified literature procedure.[1] Briefly, the cation precursors were heated in acetonitrile for a prolonged period, with PP13-Br precipitating as a white solid in the golden liquid upon cooling. Combining PP13-Br and Li-TFSI as aqueous solutions causes precipitation of a dense (> 1 g/mL), mildly viscous colourless liquid within an hour of mixing, and extraction with dichloromethane (DCM), washing with ultrapure water, and drying *in vacuo* results in the PP13-TFSI ionic liquid used for electrochemical experiments (Scheme S1).[1]

Scheme S1 Synthesis of cation precursor PP13-Br and the ionic liquid PP13-TFSI from metathesis with Li-TFSI.

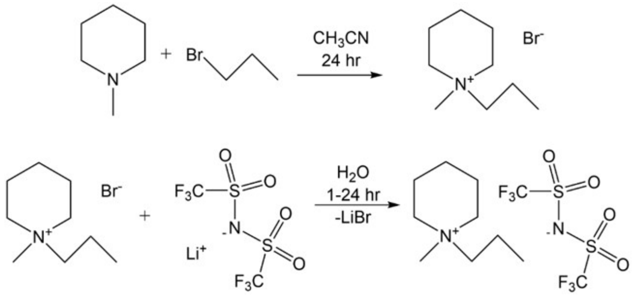

Synthesis of PP13-Br cation precursor

In a 500 mL round bottom flask 15 mL 1-bromo propane, 20 mL N-methyl piperidine and 160 mL acetonitrile were heated to 70 °C for 2.5 hours with an air condenser open to air until solid began forming, then nitrogen was supplied for the remainder of the synthesis. At the 4-hour mark, aluminum foil was wrapped around the flask. The next day at the 23-hour mark, the solid had prevented stirring. This was filtered through a coarse glass frit and rinsed with 50 mL acetonitrile in 4 aliquots, transferred to a round bottom flask and dried at 42 °C under vacuum. A second crop was harvested by concentrating the solution until solid formed, followed by cooling to 0 °C and filtering/rinsing as before. The third crop of crystals came from completely drying the filtrate to a yellow paste, triturating with 10 mL acetonitrile, then filtering and rinsing. The filtrate was disposed of, and the yield for each crop was 16.2, 8.2, and 4.9 g (44, 22, 13 % yield, total 79%) in succession after drying *in vacuo*. By ^1H NMR, each crop was pure PP13-Br with a trace of acetonitrile or water.

First Synthesis of PP13-TFSI

A solution of PP13-Br (0.9566 g) in 6 mL D.I. H_2O was slowly added to a solution of Li-TFSI (1.2362 g) in 6 mL D.I. H_2O with rapid stirring. And additional 2×2 mL D.I. H_2O was used to completely transfer the PP13-Br solution and left to stir over night. Upon initial addition, the solution of Li-TFSI

became cloudy and translucent. Ceasing stirring allowed a biphasic layer to form with PP13-TFSI on the bottom with an aqueous top layer, which was decanted off. Rinsing the bottom layer with 3×5 mL D.I. H_2O with ~10 min stirring, settling, and decanting each time was followed by dissolution of PP13-TFSI in 6 mL DCM which was similarly rinsed with 3×5 mL D.I. H_2O. The next day, the organic layer was dried over $MgSO_4$ and filtered through a diatomaceous earth pipette filter, rinsing 2× with ~3 mL $MgSO_4$ dried DCM. The final solution was dried *in vacuo* at 105 °C for 14 hours and the ionic liquid was pure by NMR, however when water was present, a shift was noticed clearly by ^{1}H NMR spectroscopy for the two -CH_3 groups with the shifted peaks disappearing with water loss. This indicates a strong interaction such as some sort of clustering or hydrogen bonding occurring between the ionic liquid and water.

Second Synthesis of PP13-TFSI

A solution of 5.4601 g PP13-Br in 15 mL H_2O was added to a solution of 7.0475g LiTFSI in 20 mL H_2O within a 50 mL Erlenmeyer flask, rinsed with 3×5 mL H_2O, totaling 50 mL. Stirring overnight was followed by quick settling of the layers the next day. Decanting the water was followed by addition of 15 mL $CHCl_3$ was added to extract remaining product and was separated with a pipette to the ionic liquid bottom fraction. Triplicate washing of the organic fraction with 20 mL water with stirring, settling and decanting in between was followed by drying over $MgSO_4$ for 20 minutes and filtering through diatomaceous Earth with 3×3 mL $CHCl_3$ rinses and the combined solvent was removed *in vacuo* and heated the remaining thick colorless viscous liquid was heated to o 100 °C in two separate vials. One vial was dropped in the oil bath, losing the product. The remaining vial of PP13-TFSI when dried contained 0.1 eq H_2O and weighed 4.5g.

Third Synthesis of PP13-TFSI

In a 125 mL Erlenmeyer flask 9.818 g PP13-Br dissolved in 60 mL H_2O had a solution of 12.665 g Li-TFSI in 20 mL H_2O added followed by 20 mL used to rinse the beaker 3 times into the reaction flask. Stirring for 1.5 hours, then add 20 mL $CHCl_3$ and rapidly stir biphasic mixture for 2 minutes, followed by settling until no more visible tiny bubbles of $CHCl_3$ were suspended in the water layer. Decanting the water was followed with triplicate washing the organic layer with three 25 mL portions. The organic phase was transferred to a separatory funnel with an additional 75 mL water, the organic phase washed once more and separated, dried over $MgSO_4$ for 20 min and filtered through diatomaceous Earth with 3×5 mL $CHCl_3$ rinses and the solvent was removed *in vacuo* and heated to 105 °C for 12 hours and store in a glovebox, total mass = 13.98 g (75 % yield)

Fourth Synthesis of PP13-TFSI

Two 10 mL water solutions were prepared containing 3.9630 g Li-TFSI and 3.1253 g PP13-Br. The PP13-Br solution was then added to the Li-TFSI solution, followed by 5 mL water to rinse. After 1.5 hours the biphasic mixture was allowed to settle, the aqueous phase was decanted, and the ionic liquid was washed 2× with 5 mL water by stirring for 0.5 hours. All three aqueous decantation's were

combined. The PP13-TFSI had 2×10 mL DCM added transferring to a separatory funnel with the organic layer being separated into anhydrous $MgSO_4$ for further drying. Filtration through diatomaceous Earth into a 20 mL scintillation vial afforded 4.3161 g of PP13-TFSI after drying overnight in a vacuum desiccator. All glassware and the aqueous phase were extracted with an additional 50 mL, drying over the same $MgSO_4$ after separation from the aqueous phase. Filtration through a cotton plug into a round bottom flask was followed by solvent removal *in vacuo*, with a final transfer and drying *in vacuo* in a 20 mL scintillation vial using an additional 2×3 mL DCM. Drying overnight in a vacuum desiccator afforded a second crop of PP13-TFSI weighing 1.4327 g. Combined yield = 98% (1st = 73 %, 2nd = 25%).

NMR Spectroscopy of Ionic Liquid, Precursors, and 1,4-Butanedithiol

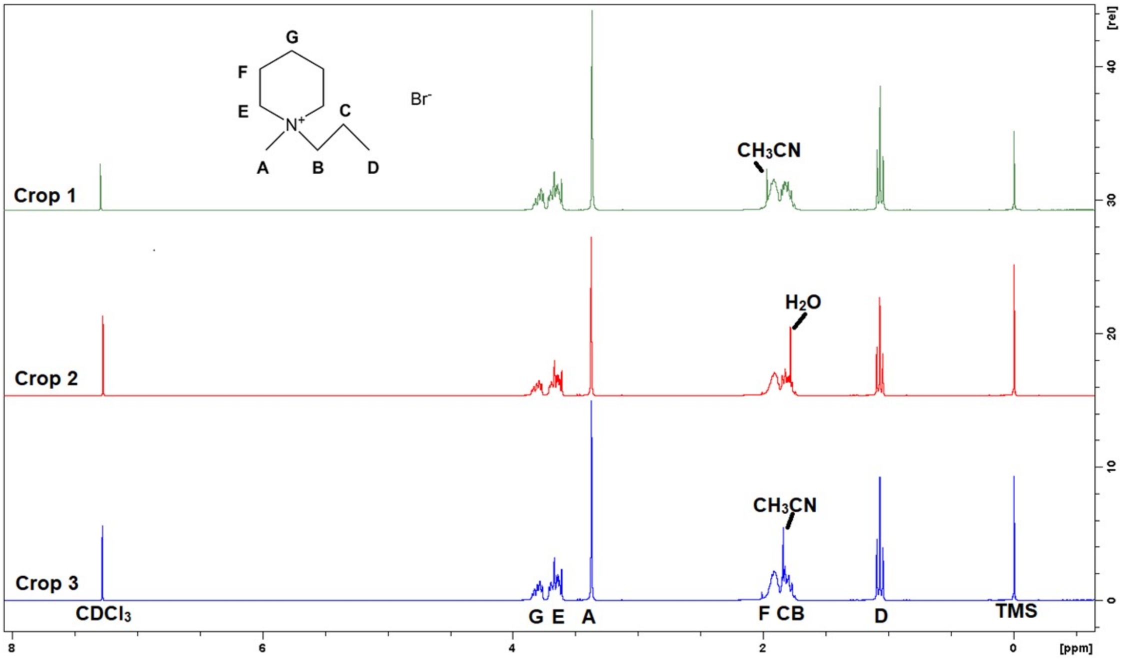

Figure S1. ^{1}H NMR spectra of [PP13][Br] in $CDCl_3$ (crops 1-3).

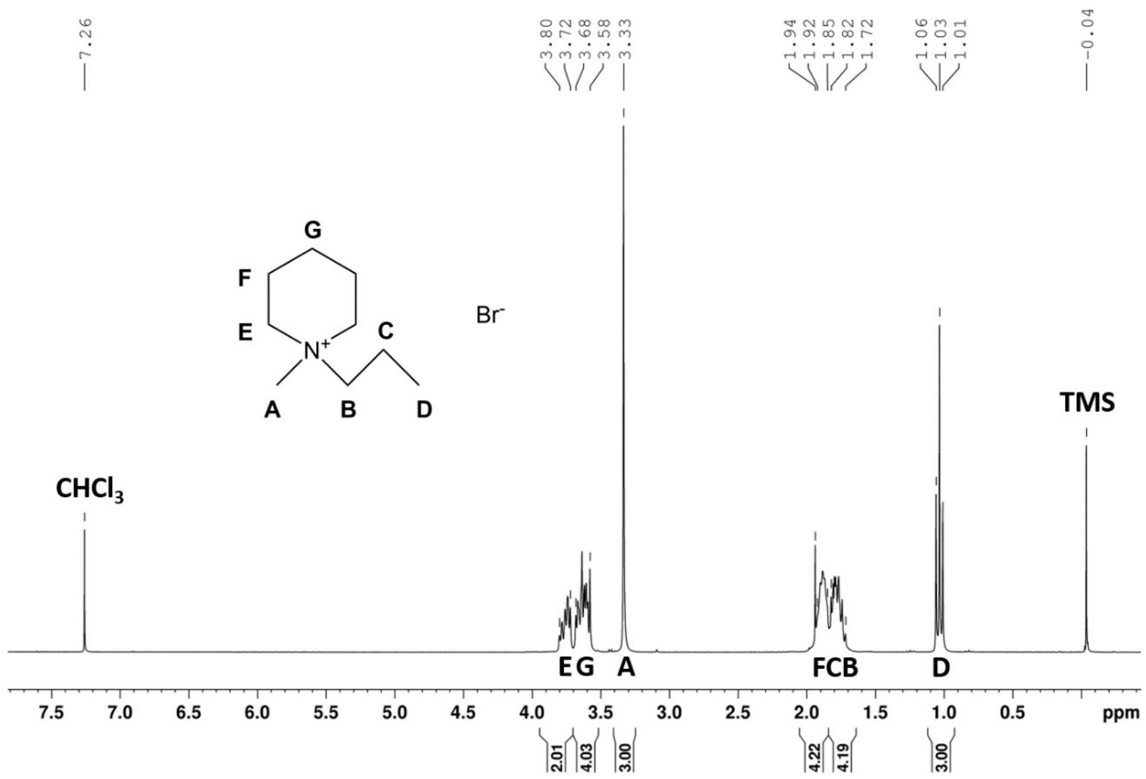

Figure S2. ^{1}H NMR spectra of [PP13][Br] in $CDCl_3$.

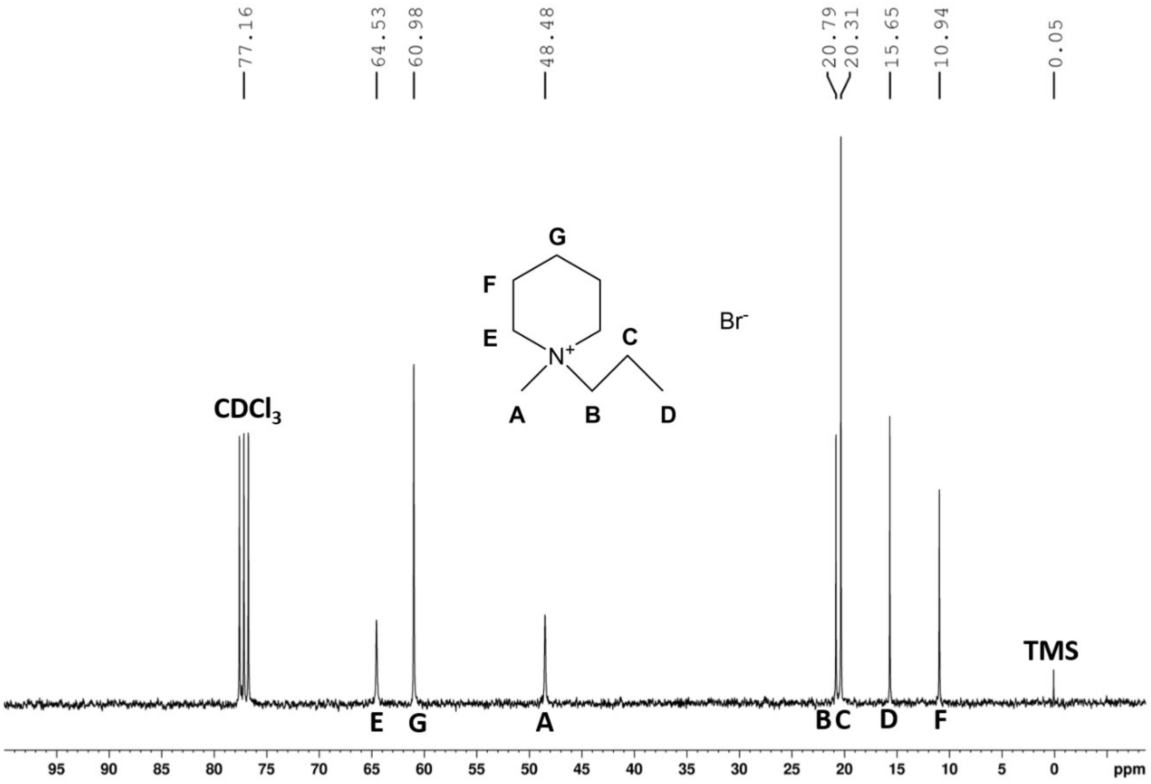

Figure S3. ^{13}C NMR spectra of [PP13][Br] in $CDCl_3$.

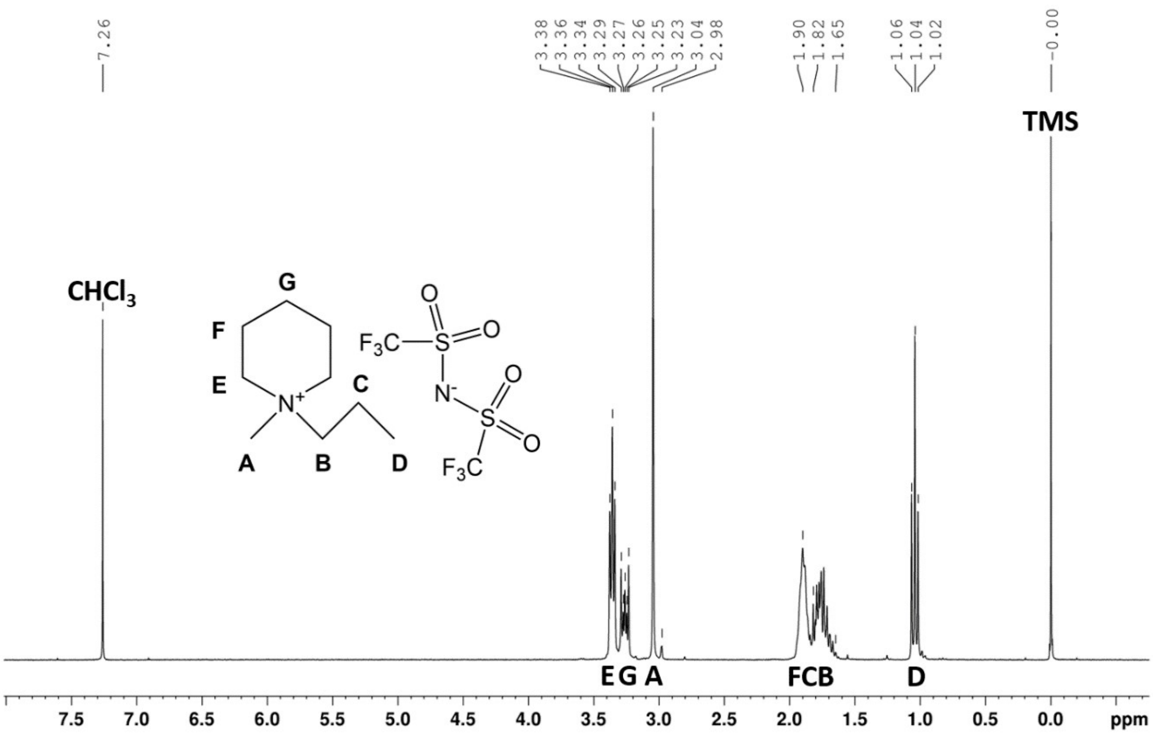

Figure S4. ^{1}H NMR spectra of [PP13][TFSI] in $CDCl_3$.

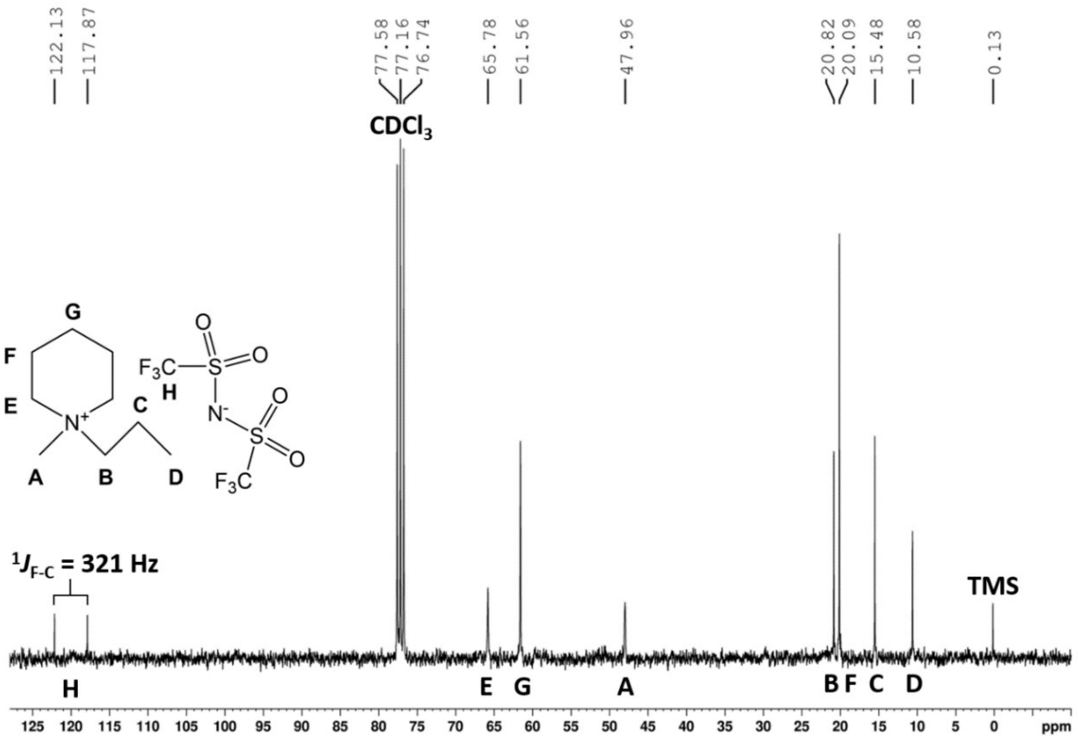

Figure S5. ^{13}C NMR spectra of [PP13][TFSI] in $CDCl_3$.

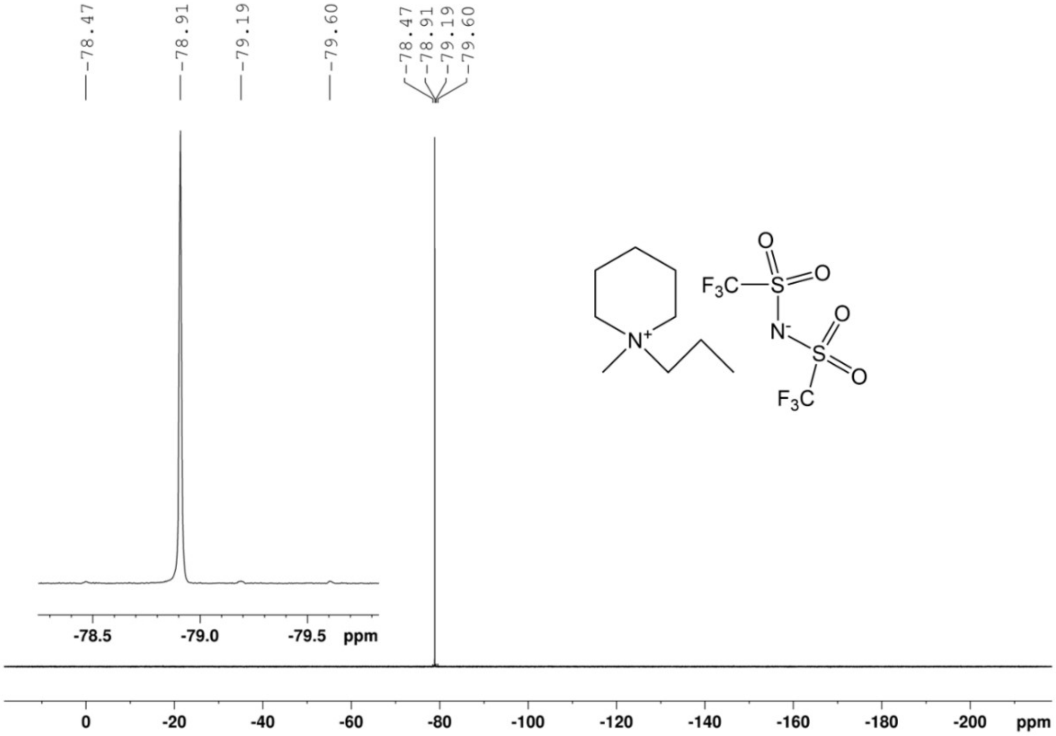

Figure S6. ^{19}F NMR spectra of [PP13][TFSI] in $CDCl_3$.

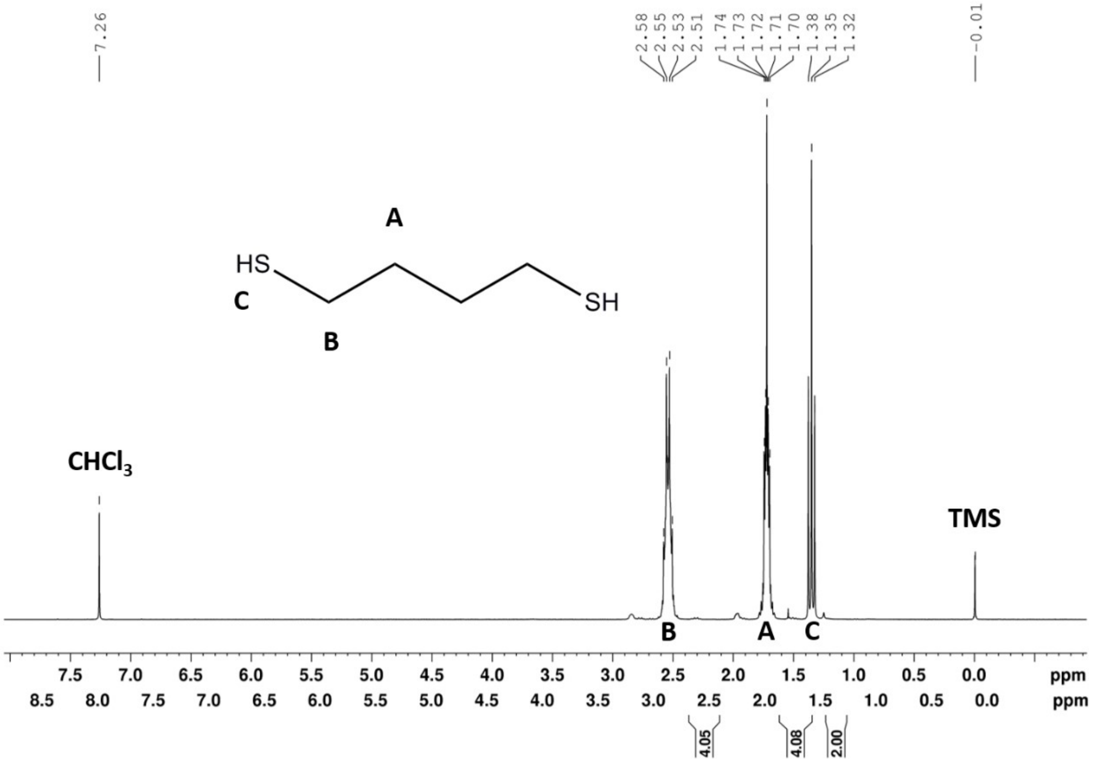

Figure S7. ^{1}H NMR spectra of 1,4-butanedithiol in $CDCl_3$.

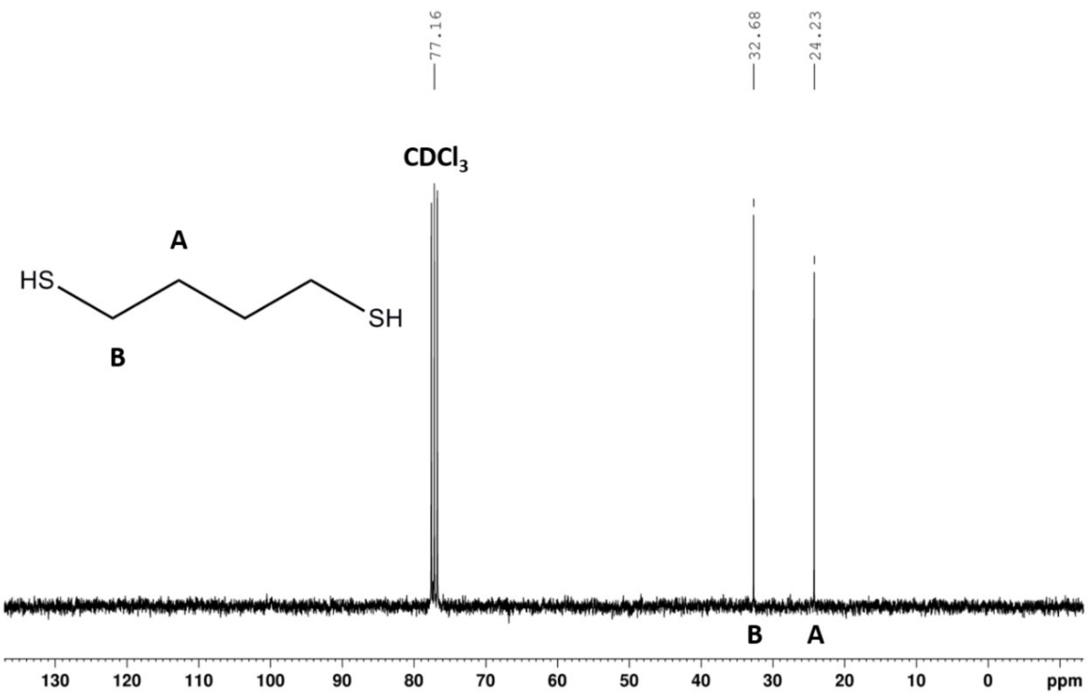

Figure S8. ^{13}C NMR spectra of 1,4-butanedithiol in $CDCl_3$.

Molybdenum Precursor

Synthesis Details

First synthesis of the Molybdenum Precursor – A1

To a 250 mL round bottom flask, 0.578g MoO_3 and ~75 mL ethylene glycol was heated under nitrogen to 194 °C for 1 hour with rapid stirring. The suspensions colour progressed from colourless with mint green powder to a dark turquoise solution at 180 °C as dissolution began, eventually turning into black at 190 °C, then to golden-brown after 40 minutes at 194 °C, and finally appearing dark reddish brown with a fine black precipitate after 1 hour at 194 °C. The crude reaction mixture was briefly exposed to air due to not using a Schlenk flask to provide N_2 flow during sampling for solvent compatibility and water exposure tests. This was allowed to sit under nitrogen for four days and then a vacuum distillation at 110 °C for 3 hours was done to remove most of the ethylene glycol, leaving a golden green paste that was extracted using 2×30 mL dichloromethane and the solution was filtered through a coarse glass frit quickly to minimize air exposure. The combined dichloromethane extracts were concentrated *in vacuo* affording a thick golden viscous liquid. A valve-less gas adapter was used while concentrating the solution and was swapped with a valved adapter and sealed under vacuum to transfer into the glovebox. The valve-less adapter had some viscous liquid residue on it, which took on a red colour upon air exposure, turning a rich blue overnight, indicating instability upon exposure to air. The sealed flask produced colourless crystals (C1a) from a gold viscous liquid. 44.5 mg of off-white crystals with a small amount of red clumps were isolated by rinsing the crude mixture with THF in a glass frit, resulting in a yield of 4.4% of $Mo^{VI}O_2(OC_2H_4OH)_2$, the identity of which was determined by single crystal X-ray diffraction, supported by elemental analysis Concentrating the THF/ethylene glycol filtrate *in vacuo* afforded a golden solution

(B1a).

Analytical Calc. for $C_4H_{10}MoO_6$: C: 19.21% H: 4.03% N: 0.00% Found: C: 19.23% H: 4.09% N: 0.00%

Second Synthesis of the Molybdenum Precursor – A2

In a 250 mL Schlenk flask, 0.800 g MoO_3 and 125 mL ethylene glycol was heated to 194 °C for 1 hour under N_2 resulting in the same colour change the first synthesis, turning greenish blue at ~184 °C followed by a golden-brown/black. Upon cooling to 80 °C, a vacuum distillation was started, and heating was increased to 110-115 °C for 3 hours, which was stopped and left to sit under N_2 until the next day and resumed for an additional 5 hours until about 3 mL of golden viscous liquid was present with a green paste. 1x100 mL dichloromethane was added and left to stir for 1 hour. The filtration utilized a two ended frit with a 1.5 cm pad of diatomaceous earth on the side of the crude extract and was filtered into a second 250 mL Schlenk flask with positive pressure from the reaction flask. The golden extract was reduced to ~ 3 mL golden viscous liquid that was sealed under vacuum and transferred to the glovebox. This was then transferred to a 20 mL vial with ~10 mL THF and the solvent was removed *in vacuo*. The residue had hexane added which did not dissolve the solid, so it was removed, and toluene was added with subsequent removal *in vacuo*. 2×5 mL THF was used to extract the solid through a celite filled pipet filter, affording a filtrate which, upon cooling, was a thick golden viscous liquid with yellow crystals (C2a) identified to be $(Mo^{V}O(OC_2H_4OH)(\mu\text{-}OC_2H_4OH))_2(\mu\text{-}OC_2H_4)$ by SC-XRD. Filtering and rinsing the solid with THF 2× and drying *in vacuo* afforded 374 mg of a yellow opaque solid (D2a) and removal of THF *in vacuo* from the filtrate left a thick green viscous liquid (B2a). The residue on the diatomaceous earth pad from the dichloromethane filtration was rinsed with water and dried *in vacuo* affording crystals of $Mo^{VI}O_2(OC_2H_4OH)_2$ (C2b, identified by SC-XRD) in a dark green viscous liquid.

Analytical Calc. for $C_{12}H_{28}Mo_2O_{11}$: C: 26.68% H: 5.22%. Found: C: 27.59% H: 3.19%.

Fourth Synthesis of the Molybdenum Precursor – A4

To a 250 mL round bottom flask 0.466 g MoO_3 and 50 mL ethylene glycol were heated in a distillation set up under vacuum to 170 °C removing ~6 mL colourless liquid prior to heating. The flask was filled with N_2 and heated to 194 °C, with the colour changing from greenish blue at 170 °C to golden-brown at 194 ° C. The temperature was increased from 194 °C to 215 °C over 1 hour resulting in a black suspension in brown liquid that was heated for an additional 2 hours, followed by cooling with vacuum distillation at 130 °C, then the black remaining solid was heated under vacuum at 160 °C until dry. The flask was transferred to the glovebox and transferred into a 20 mL scintillation vial yielding 0.491 g of a lustrous, metallic black solid (D4a). The residue in the flask was dissolved in water and transferred to a 20 mL vial in a -18 °C freezer with atmospheric exposure, resulting in a blue solution (B4a) forming very tiny blue crystals (C4a) that rapidly melted/dissolved upon warming. By SC-XRD, the blue crystal was identified as a highly hydrated $Mo_{12}O_{40}X$ cluster with an unknown central atom or alkali/alkaline earth

cations between the Mo/O/X clusters. Drying the blue solution *in vacuo* afforded a dark blue amorphous solid (D4b).

Seventh Synthesis of the Molybdenum Precursor – A7

In an apparatus composed of a Schlenk reaction flask and round bottom collection flask with vacuum distillation adaptors, 282 mg of MoO_3 and 35 mL ethylene glycol were heated to 194 °C under nitrogen flow, forming dark golden solution in 1.5 hours after reaching temperature. Vacuum distillation was conducted for ~2 hours at 125 °C on the heating mantle with aide from a heat gun (upper walls of flask not touched by heating mantle) to a green paste. Triplicate extraction with 3×15 mL dichloromethane, filtering through a coarse frit, and removal of solvent *in vacuo* afforded a golden viscous liquid (B7a). Rinsing with THF afforded 59 mg of a yellow microcrystalline solid.

Eighth Synthesis of the Molybdenum Precursor – A8

In an apparatus composed on two Schlenk flasks set up for distillation, 128 mg of MoO_3 and 20 mL ethylene glycol were heated to 150 °C under nitrogen flow, forming a homogenous golden solution in 2.5 hours after reaching temperature. Cooling afforded no precipitate when sealed overnight. Vacuum distillation was conducted for ~4 hours between 100-120 °C in an aluminum block on a hotplate with aide from a heat gun (upper walls of flask not touched by heating mantle) to a final volume of 1 mL gold viscous liquid (B8a). Addition of 10 mL 95 % ethanol afforded a rich blue solution (B8b) and filtration followed by rinsing with 10 mL diethyl ether afforded < 5 mg residue on the filter paper. The filtrate was dried *in vacuo* affording a dark blue solid of unknown identity weighing 181 mg (D8a).

Table S1 Reagents and reaction conditions associated with the 8 molybdenum precursor syntheses.

Rxn.	MoO3 mass (g)	Ethylene glycol (EG) volume (mL)	Concentration (g MoO3/mL ethylene glycol)	Reaction time (min)	Temperature (± 2 °C)
A1	0.578	75	0.008	60	194
A2	0.800	125	0.006	60	194
A3	0.345	40	0.008	60	194
A4	0.466	50	0.009	120	215
A5	0.544	70	0.008	60	194
A6	0.286	35	0.008	60	194
A7	0.128	20	0.006	90	194
A8	0.282	35	0.008	150	150
Mean	0.429	56	0.008	80	191
St.Dev	0.211	33	0.001	35	18

Table S2. Synthetic conditions, work up, purification processes, and products isolated from the 8 molybdenum precursor syntheses. Volume in brackets = EG distilled prior to heat.

Rxn.	Pre-distill EG before 194 °C?	N_2 flow? Air exposure?	Solvents for purification or Workup Procedure?	Products isolated
A1	No	Yes, minor	Vacuum distill to green paste, DCM extraction, THF wash	Colourless crystals of $MoO_2(OC_2H_4(OH))_2$ and golden viscous liquid
A2	No	Yes, trace	Vacuum distill to green paste, DCM extraction, THF transfer, hexane and toluene insoluble, THF extraction	Green solid, yellow crystals from green viscous liquid, Colourless crystals of $MoO_2(OC_2H_4(OH))_2$ upon partial hydrolysis of residue
A3	Yes (~5 mL)	Yes, no	Vacuum distill, filter	Golden viscous liquid, Colourless crystals of $MoO_2(OC_2H_4(OH))_2$ after 10 months under N_2
A4	Yes (~6 mL)	Yes, no	Vacuum distill, scrape	Black solid, blue crystal of $Mo_{12}O_{40}X$ upon hydration of residue
A5	No	Yes, no	Vacuum distill, filter, rinse residue into separate container with THF	Golden-brown viscous liquid and gold/yellow crystals
A6	No (~10 mL)	Yes, no	Vacuum distill, filter, rinse residue into separate container with THF	Brown viscous liquid with black solids, red crystals
A7	No	Yes, no	Vacuum distill to green paste, DCM extraction, THF wash	Yellow crystals, gold viscous liquid
A8	No	No, no	Vacuum distill, 95% ethanol, filter, dry	Gold viscous liquid turned into blue liquid then dried to blue solid

Crystallography

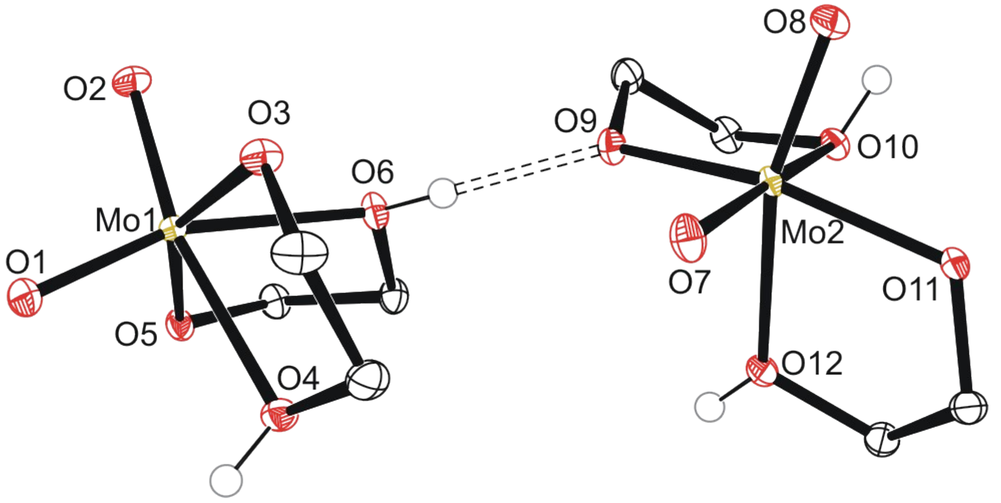

Figure S9. Molecular structure of the two molecules in the asymmetric unit cell of compound **1** participating in hydrogen bonding with anisotropic displacement ellipsoids projected at the 50% probability level. Methylene CH_2 hydrogen atoms have been omitted for clarity.

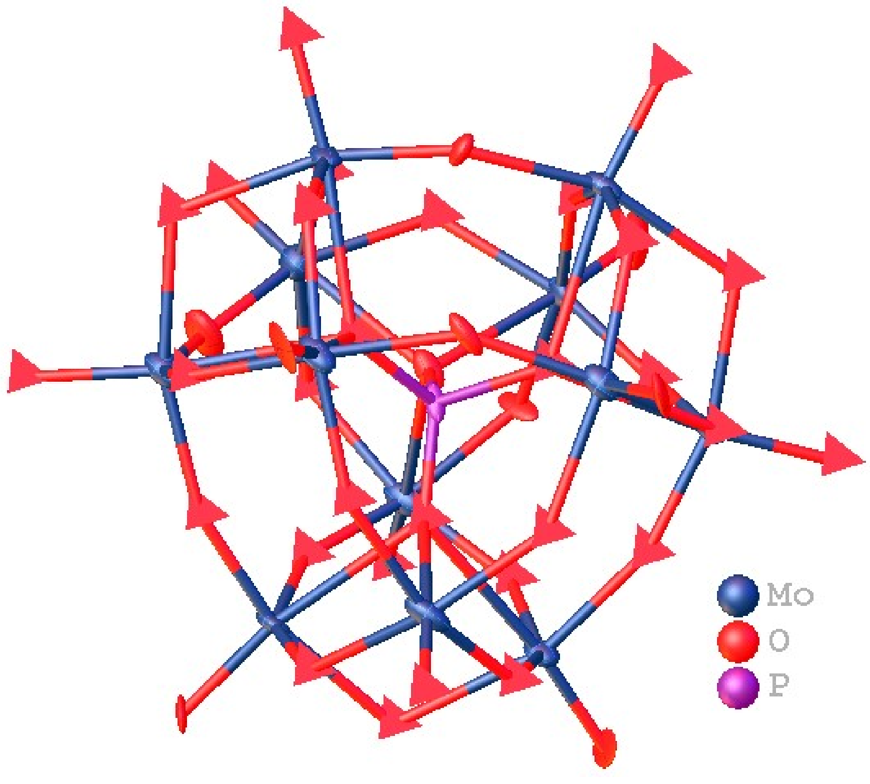

Figure S10. OLEX2 screenshot[2] showing the large anionic heteroatom-containing cluster $[Mo_{12}O_{40}X]^{-n}$ where X is best modelled as phosphorus, suggesting this is a tri- or pentaanionic cluster with a lot of water of solvation. This is the dataset derived from a single isolated blue crystal (**C4a**) from hydrolysis of **D4a** residue, filtration, and freezing at -18 °C. Co-crystallized solvent and cations could not be modelled effectively and were removed for this image. Poor data resulted in only evidence of this cluster forming with inconclusive structural/compositional determinations.

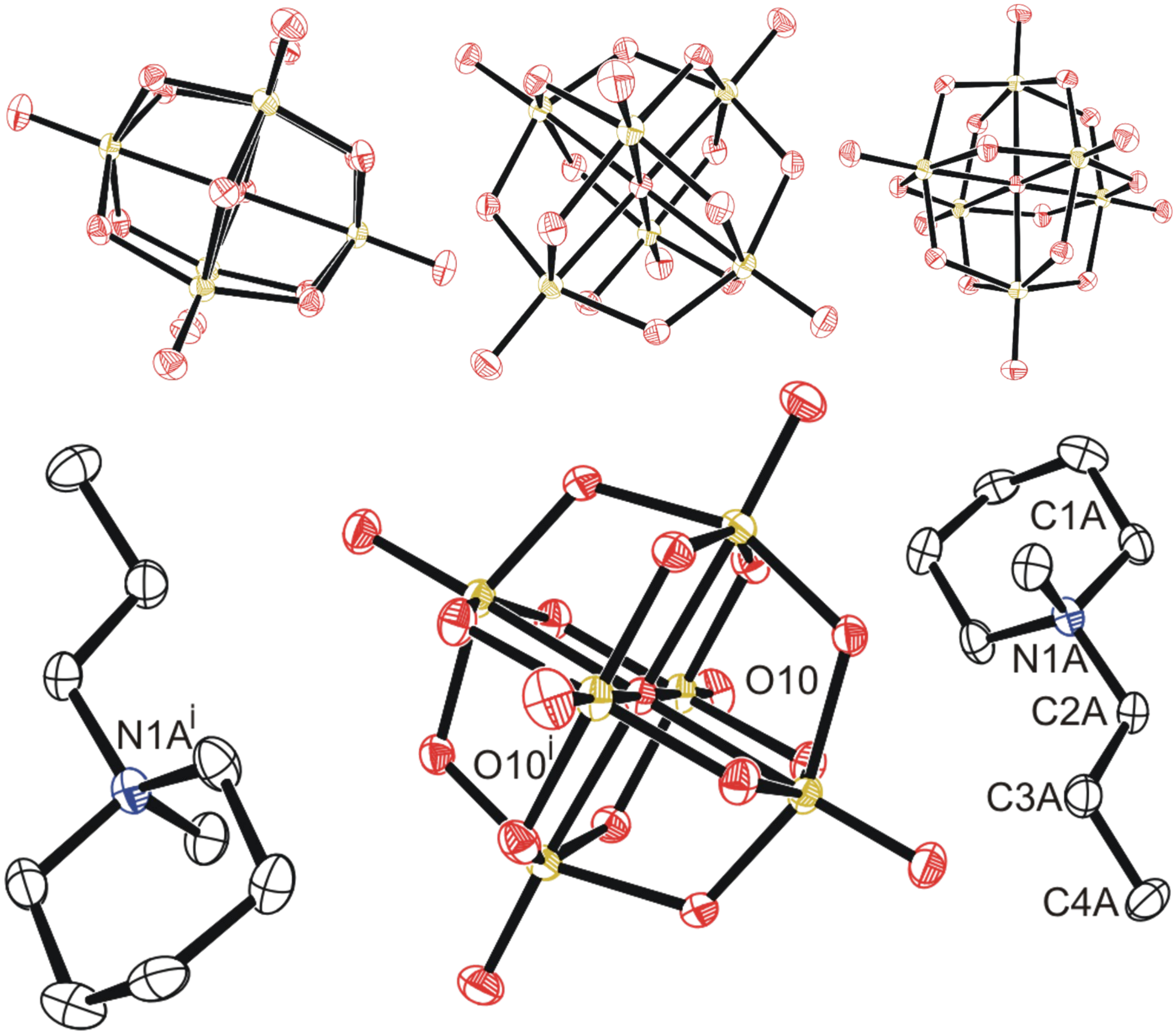

Figure S11. Molecular structure of the single yellow crystal (**C3b**) compound **5** with anisotropic displacement ellipsoids projected at the 50% probability level. Hydrogen atoms have been omitted for clarity. From top left to right represent views of the dianion $[Mo_6O_{19}]^{2-}$ down the *a*, *b*, and *c* axes respectively. The bottom image represents the complete structure of compound **5**; $[PP13]_2[Mo_6O_{19}]$. Oxygen = red, molybdenum = yellow.

Table S3. Data derived from single crystal X-ray diffraction of crystals of compounds **1-5**.

Compound	Compound **1**	Compound **2**	Compound **3**	Compound **4**	Compound **5**
Chemical formula	$C_4H_{10}MoO_6$	$C_8H_{18}Mo_2O_{10}$•C_4H_8O	$C_{16}H_{36}Mo_4O_{20}$	$C_{24}H_{54}Mo_8O_{32}$•2($C_2H_6O_2$)	[Mo_6O_{19}]•2[$C_9H_{20}N$]
Formula Mass	250.06	538.21	932.21	1746.32	1164.16
Crystal system	Tetragonal	Orthorhombic	Orthorhombic	Triclinic	Monoclinic
a/Å	9.46450(10)	16.7363(3)	34.2851(19)	8.5318(4)	9.4347(8)
b/Å	9.46450(10)	24.6210(5)	9.7131(5)	11.0284(5)	16.0288(12)
c/Å	17.3160(4)	8.8062(2)	8.4881(4)	14.5245(7)	10.9136(9)
α/°	90	90	90	81.706(2)	90
β/°	90	90	90	77.862(2)	101.838(3)
γ/°	90	90	90	69.992(2)	90
Unit cell volume/Å^3	1551.11(5)	3628.72(13)	2826.7(3)	1251.55(10)	1615.3(2)
Temperature/K	100.0	125.0	125	125.0	125.0
Space group	$P4_1$	Fdd2	$Pna2_1$	*P***Error!**	$P2_1/n$
No. of formula units per unit cell, *Z*	8	8	4	1	2
Radiation type	MoKα	MoKα	MoKα	MoKα	MoKα
Absorption coefficient, μ/mm^{-1}	1.675	1.436	1.819	2.040	2.339
No. of reflections measured	110585	26440	281817	126602	55716
No. of independent reflections	12760	3835	13707	10104	6194
R_{int}	0.0334	0.0568	0.0390	0.0407	0.0377
Final R_1 values ($I > 2\sigma(I)$)	0.0208	0.0309	0.0655	0.0219	0.0332
Final $wR(F^2)$ values ($I > 2\sigma(I)$)	0.0508	0.0448	0.1327	0.0528	0.0704
Final R_1 values (all data)	0.0223	0.0485	0.0682	0.0267	0.0394
Final $wR(F^2)$ values (all data)	0.0518	0.0487	0.1339	0.0554	0.0731

Table S4. Hydrogen bonding interactions observed for compounds **1**-**4**.

Donor	H	Acceptor	d(D-H)/Å	d(H-A)/Å	d(D-A)/Å	D-H-A/°
Compound **1** – $MoO_6C_4H_{10}$						
O4	H4	O3[1]	0.851(13)	1.852(14)	2.7000(18)	174(4)
O6	H6	O9	0.854(13)	1.836(19)	2.6406(17)	156(4)
O10	H10	O5[2]	0.855(13)	1.808(15)	2.6517(16)	169(4)
O12	H12	O11[3]	0.76(4)	1.75(4)	2.5047(17)	171(4)
Compound **2** – $Mo_2O_{10}C_8H_{18}$•(OC_4H_8, THF)						
O5	H5	O2[4]	0.89(3)	1.93(3)	2.814(2)	175(4)
Compound **3** – $Mo_4O_{20}C_{16}H_{36}$						
O5	H5	O4	0.873(13)	1.83(3)	2.582(9)	142(3)
O5	H5	O18[5]	0.873(13)	2.67(6)	3.273(9)	127(6)
O17	H17	O11	0.84	1.79	2.561(10)	151.7
C5	H5B	O18[5]	0.99	2.52	3.268(15)	132.6
C6	H6B	O14B[6]	0.99	2.32	3.19(4)	147
C8	H8A	O1A[7]	0.99	2.08	2.91(4)	140.2
C13	H13A	O10[8]	0.99	2.52	3.203(12)	126.1
C14	H14B	O10[8]	0.99	2.42	3.066(12)	122.5
O1A	H1A	O7	0.84	2.23	2.67(4)	112.9
O14A	H14C	O15	0.84	1.86	2.685(13)	168.7
Compound **4** – $Mo_8O_{32}C_{24}H_{54}$•2($C_2H_4(OH)_2$, ethylene glycol)						
O12	H12	O8	0.74(3)	1.95(3)	2.686(2)	174(4)
O13	H13	O17	0.94(3)	1.62(3)	2.552(2)	170(3)
O16	H16	O3[9]	0.77(3)	1.73(3)	2.4891(19)	174(4)
O17	H17	O12	0.77(3)	1.89(3)	2.657(2)	171(4)
O18	H18	O4[10]	0.773(17)	1.963(18)	2.720(2)	166(3)
Symmetry operators for relevant hydrogen bonding interactions indicated with superscripts on acceptor atoms: [1]1+Y,2-X,-1/4+Z; [2]2-X,1-Y,1/2+Z; [3]+Y,2-X,-1/4+Z; [4]3/4-X,1/4+Y,-1/4+Z; [5]+X,+Y,1+Z; [6]1-X,2-Y,1/2+Z; [7]1/2-X,-1/2+Y,1/2+Z; [8]+X,-1+Y,-1+Z [9]1-X,1-Y,1-Z; [10]-1+X,1+Y,+Z						

Compound **1** has four hydrogen bonding interactions between mono-deprotonated ethylene glycol ligands. The asymmetric unit includes the O6-H6…O6 H-bond, while hydrogen bonds also occur between O12-H12…O11 across the symmetry element +Y,2-X,-0.25+Z, O4-H4…O3 across 2-X,1-Y,0.5+Z, and O10-H10…O5 across 2-X,1-Y,0.5+Z. Compound **2** has one intramolecular hydrogen bond between O6-H6…O3 across the symmetry element 0.75-X,0.25+Y,-0.25+Z. Compound **3** has three disordered ethylene glycol units in a 54:46, 50:50, and 63:37 ratio. Compound **3** has five hydrogen bonding interactions with one (O14B-H14D…O20) across the symmetry element 1-X,1-Y,-0.5+Z. The other four interactions occur between O5-H5…O4, O14A-H14C…O15, O17-H17…O11, and O1B-H1B…O7. Compound **4** has one disordered ethylene glycol methylene fragment in a 72:28 ratio. Co-crystallized ethylene glycol solvent participates extensively in hydrogen bonding within **4**. Intermolecular bonding

occurs first from O13-H13…O17 from the compound to the ethylene glycol, and the same hydroxy group donates back to the compound via O17-H17…O12 with further intramolecular bonding from O12-H12…O8. The other end of the ethylene glycol is hydrogen bound to an adjacent molecule of **4** as O18-H18…O4 across the symmetry element -1+X,1+Y,+Z. Additional intramolecular hydrogen bonding between each molecule of **4** from O16-H16…O3 across 1-X,1-Y,1-Z exists, holding the symmetry equivalent halves of the structure together among other bonds.

Ratio of Molybdenum and Oxygen

Table S5 shows relevant Mo:O ratios observed within six SC-XRD samples showing the ratio of Mo:O drops as the colour of the viscous liquid the crystal was isolated from (and the crystal itself) darkens. Compound **1** isolated from **C1a**, **C2b**, and **C3a** has the highest Mo:O ratio of 1:6, while a drop is observed for the dimeric **2** from **B2a** green viscous liquid (1:5.5), the dimer-polymer **3** from **B5a** golden-brown viscous liquid (1:5), and the larger cluster **4** observed from **B6a** brown viscous liquid with either 1:4 as just the cluster, or 1:4.25 accounting for ethylene glycol solvation. Compounds **5** and **6** feature the lowest Mo:O ratios of 1:3.17 and 1:3.33 for the molybdenum oxide clusters $[Mo_6O_{19}]^{-2}$ and $[Mo_{12}O_{40}X]^{-n}$, respectively.

Table S5. Mo:O ratios for each of the isolated crystalline samples from molybdenum precursor synthesis (**1-4**), electrochemical experiments (**5**), and hydrolysis (**6**).

Compound	Mo:O ratio in crystal, reduced Mo:O ratio
1	**1:6**
2	2:11, (**1:5.5**)
3	4:20, (**1:5**)
4	8:32 (+ 4 co-crystallized solvent), (**1:4** cluster, **1:4.25** compound)
5	6:19, (**1:3.17**)
6	12:40, (**1:3.33**)

Electrochemistry, Electrodeposition, and SEM Analysis

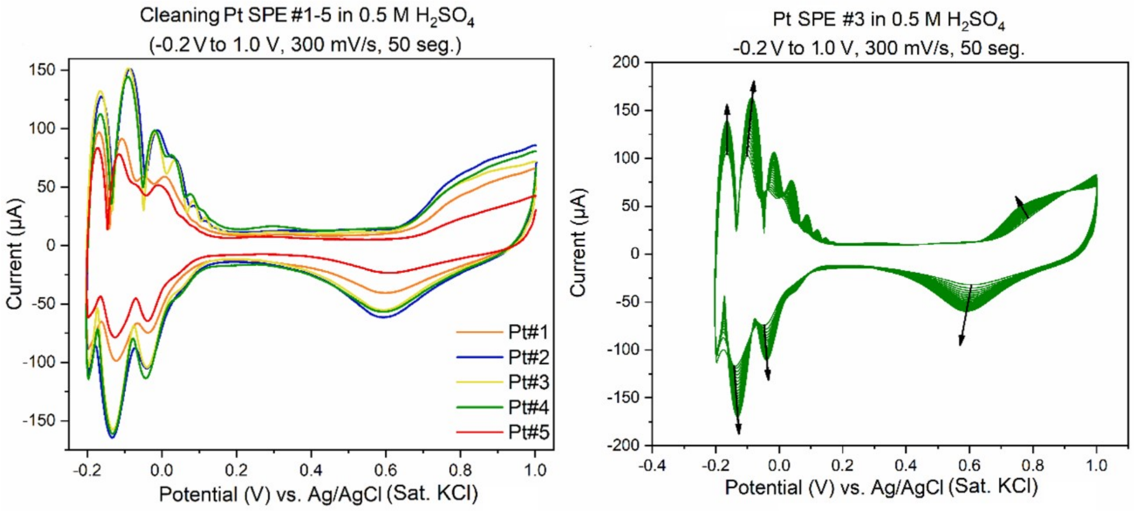

Figure S12. Final cyclic voltammogram of Pt SPE #1-5 (left) and 25 cycles of Pt SPE #3 (right) immersed in 0.5 M H_2SO_4 within a window of -200 to 1000 mV vs. Ag/AgCl in sat. KCl gel. Difference in current indicates a difference in available surface area to transfer or receive more electrons, registering as a greater magnitude of total current. Current also increases within increasing scans from cleaning the surface of the electrode, revealing more of the platinum surface to adsorb hydrogen (- V) or oxygen (+ V).

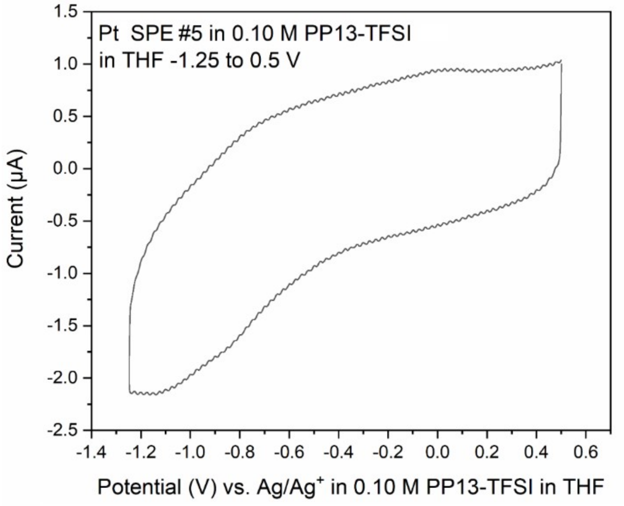

Figure S13. Double layer cyclic voltammogram showing the electrochemical window for the Pt SPE (-1.25 to 0.5 V) within the 0.1 M PP13-TFSI in THF supporting electrolyte solution (CE = Pt, RE = Ag/Ag$^+$ in 0.1 M PP13-TFSI). Sweep rate = 50 mV/s.

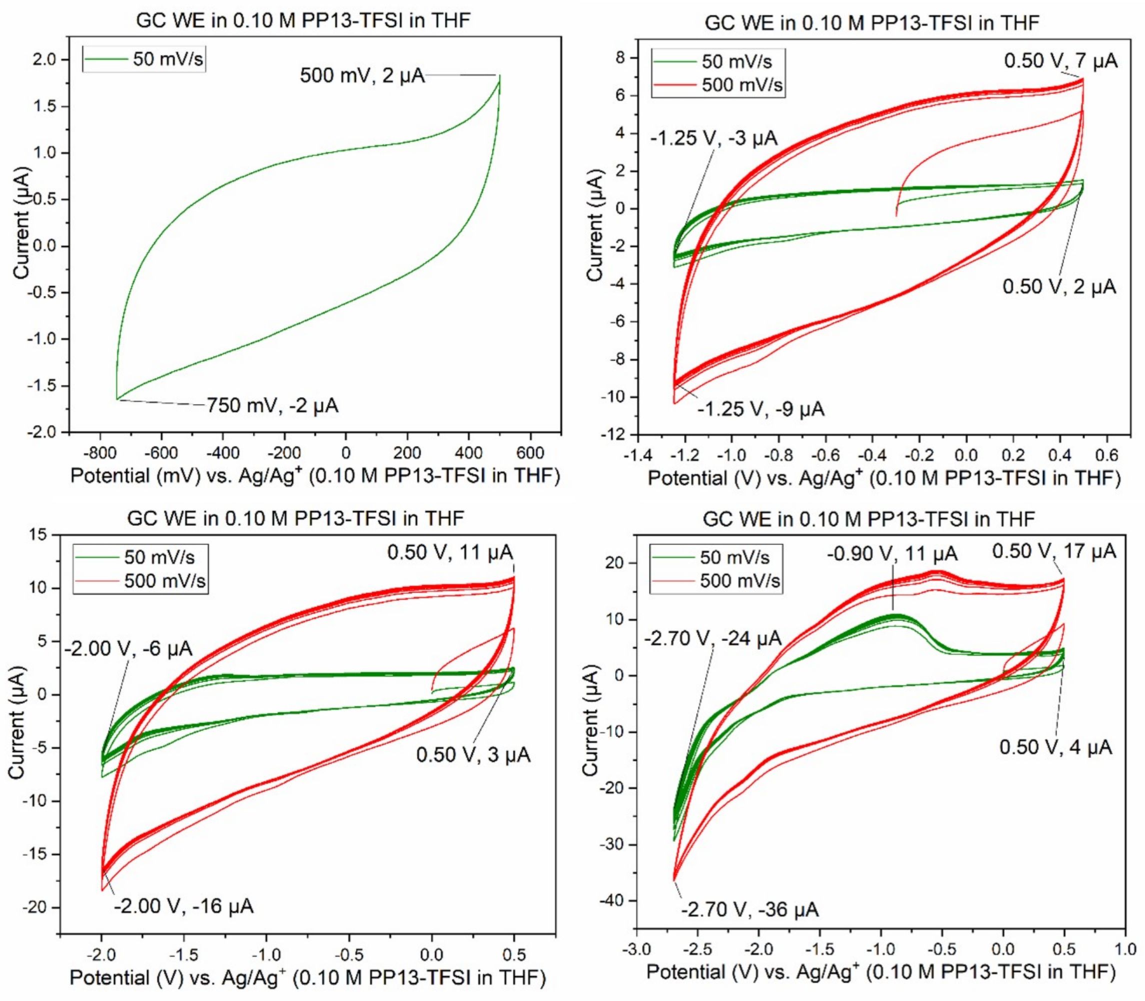

Figure S14. Electrochemical window elucidation for the 0.10 M PP13-TFSI in THF solution used for later electrochemical studies. For all but the first chromatogram, 6 cycles are shown for each sweep rate of either 50 (green) or 500 (red) mV/s.

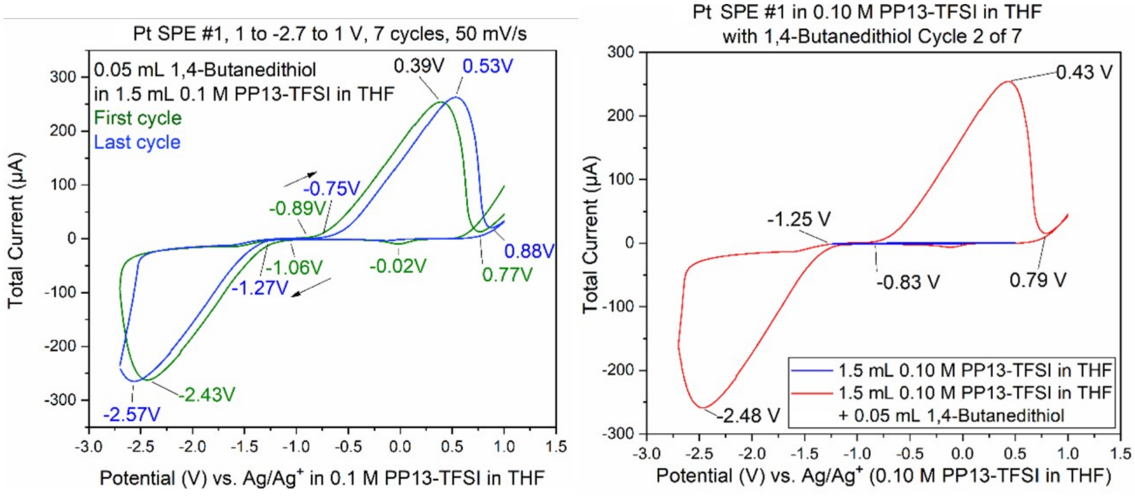

Figure S15. CV of 0.05 mL 1,4-butanedithiol added to 1.5 mL of 0.1 M PP13-TFSI in THF cycled between -2.7 V to +1.0 V at 50 mV/s. Left shows the difference between first and last cycles, while right displays the second cycle alongside a cycle in pure 0.1 M PP13-TFSI in THF.

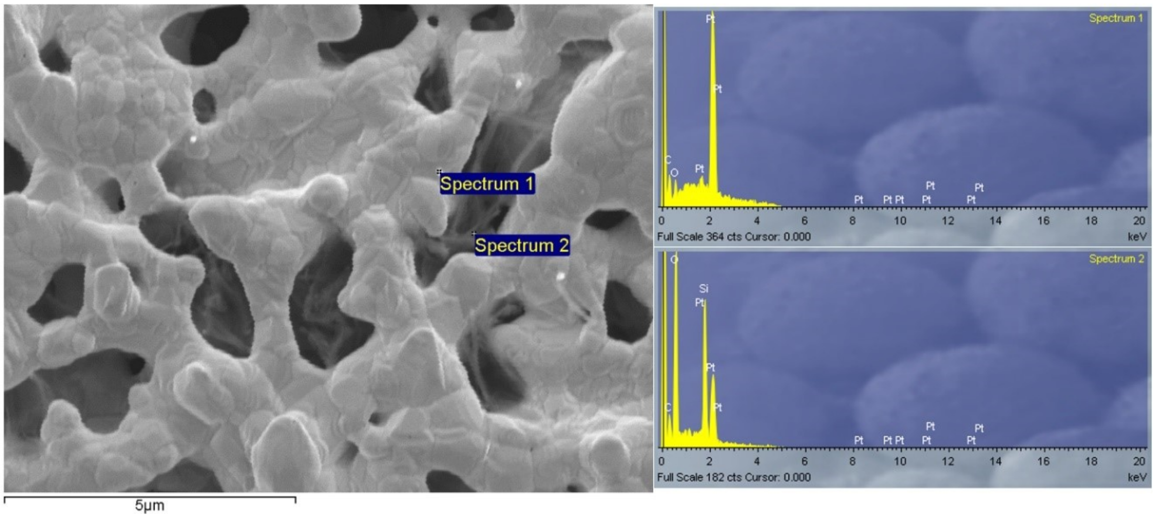

Figure S16. SEM-EDS analysis of Pt SPE #1 following electrochemistry within 1.5 mL 0.1 M PP13-TFSI in THF with 0.05 mL 1,4-butanedithiol with the corresponding SE reference image (left).

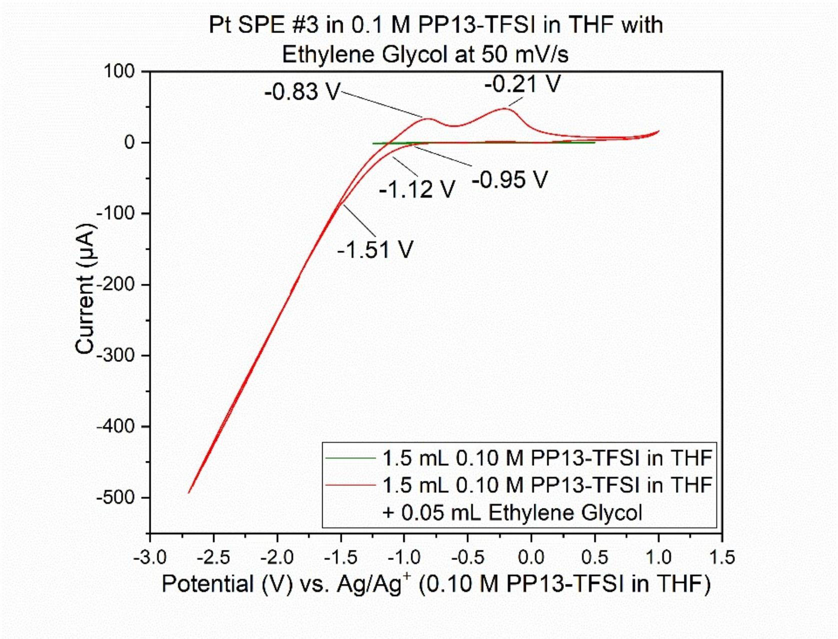

Figure S17. CV of 0.05 mL ethylene glycol added to 1.5 mL 0.1 M PP13-TFSI in THF cycled between -2.7 V to +1.0 V.

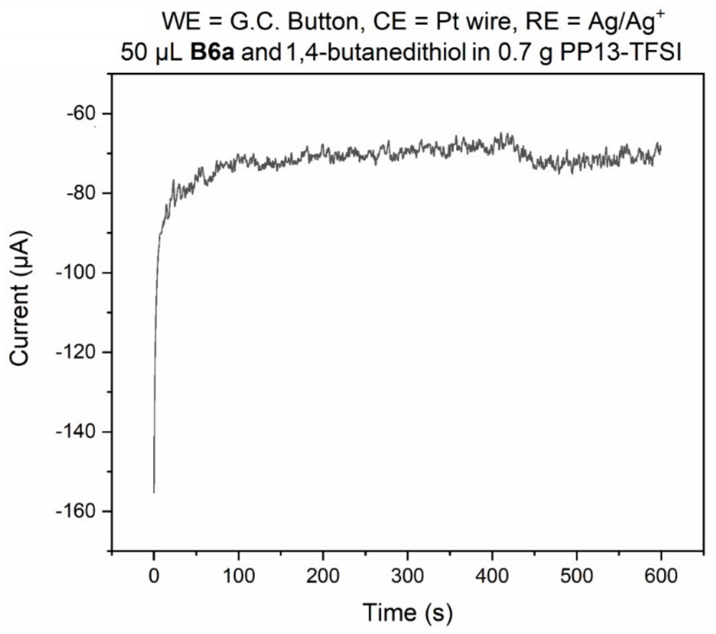

Figure S18. Chronoamperometric (CA) deposition attempt at -2.7 V for 600 seconds in a solution of 0.7g PP13-TFSI, 0.05 mL **B6a** and 0.05 mL 1,4-butanedithiol.

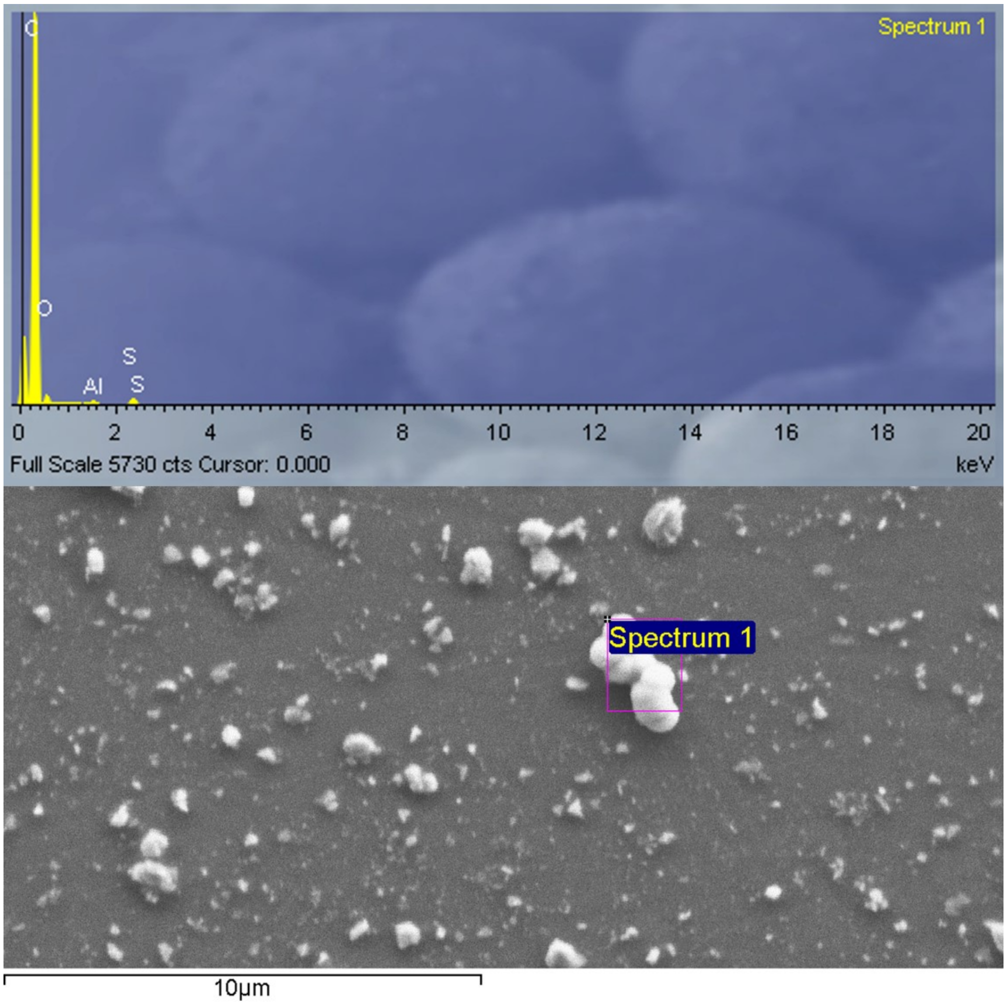

Figure S19. SEM-EDS analysis of a glassy carbon electrode following attempted chronoamperometric deposition of MoS_2. Sulfur is present within the brightest globular structures observed.

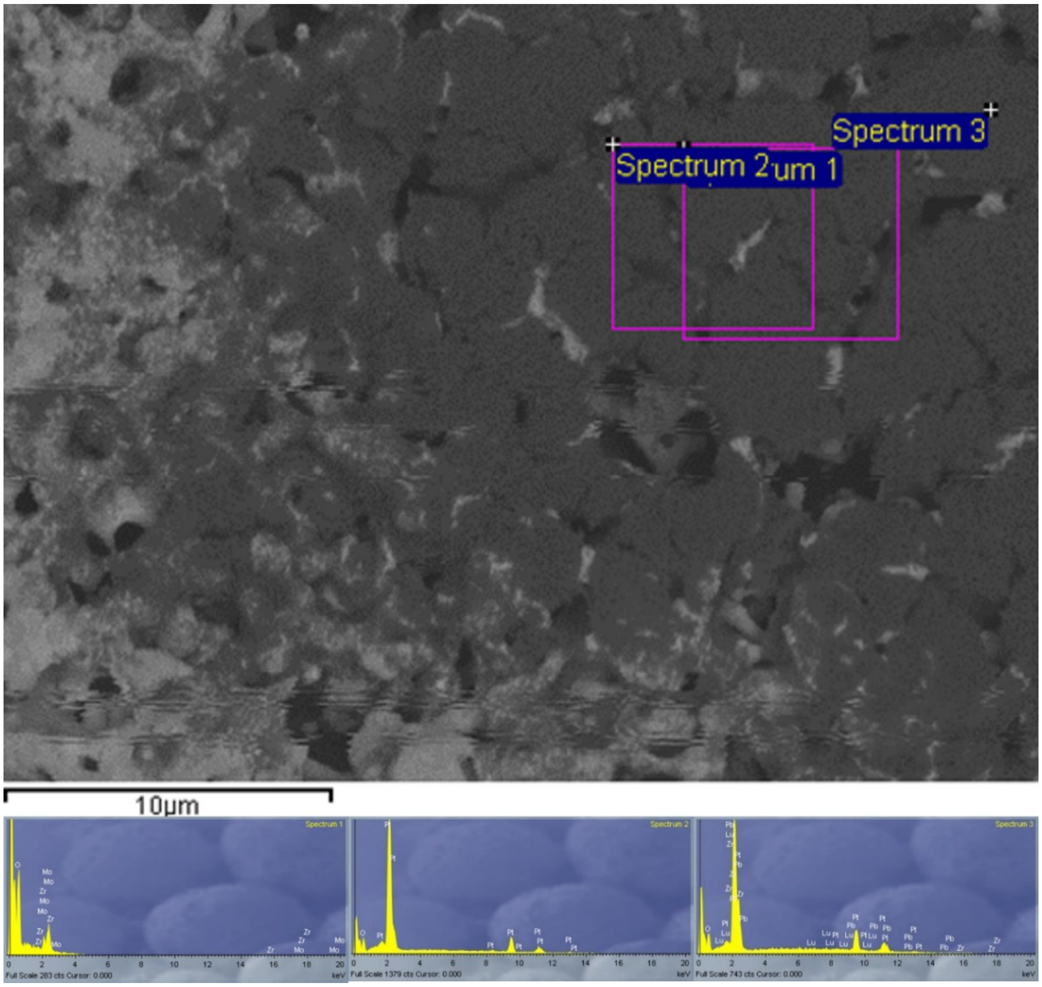

Figure S20. SEM-EDX analysis of Pt WE after CV of 0.05 mL **B6a** in 1.5 mL of 0.1 M PP13-TFSI in THF. Lu, Pb, and Zr are thought to be artifacts caused by Mo/Pt overlap.

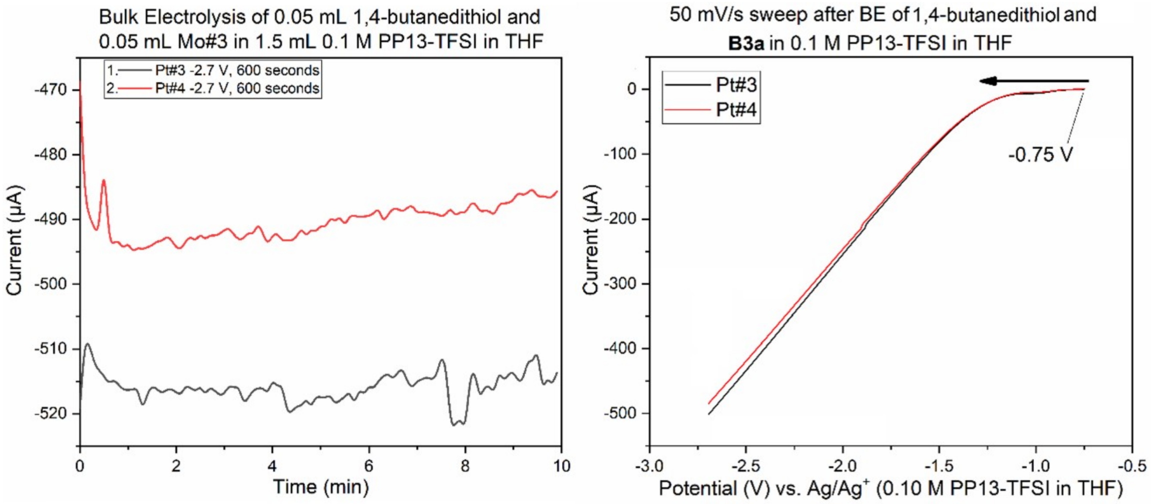

Figure S21. Two-step electrodeposition attempt involving a single solution of 1.5 mL of 0.1 M PP13-TFSI in THF which had 0.05 mL **B3a**, then 0.05 mL 1,4-butanedithiol added and left to sit for a week. The red solution was put into the electrochemical cell, and Pt SPE #3 followed by Pt SPE #4 had BE conducted at -2.7 V for 600 seconds (left) then by a single 50 mV/s sweep from -0.75 V to -2.7 V (right).

References

1. Sakaebe, H.; Matsumoto, H. N-Methyl- N-propylpiperidinium bis(trifluoromethanesulfonyl)imide (PP13–TFSI) – novel electrolyte base for Li battery. *Electrochemistry Communications* **2003**, *5*, 594-598.

2. Dolomanov, O. V.; Bourhis, L. J.; Gildea, R. J.; Howard, J. A. K.; Puschmann, H. OLEX2: a complete structure solution, refinement and analysis program. *Journal of Applied Crystallography* **2009**, *42*, 339-341.
